# Correlated electrons extend X-ray high-harmonic generation beyond the single-electron limit

**DOI:** 10.1038/s41566-026-01976-2

**Published:** 2026-08-07

**Authors:** Siyang Wang, Jieyu Yan, Alba de las Heras, Sirius Song, Aleksander Prodanov, Zhihan Wu, Luis Plaja, Dimitar Popmintchev, Tenio Popmintchev

**Affiliations:** 1 Department of Physics and Center for Advanced Nanoscience, University of California San Diego, La Jolla, CA USA; 2 Departamento de Física Aplicada, Universidad de Salamanca, Salamanca, Spain; 3 Unidad de Excelencia en Luz y Materia Estructuradas, Universidad de Salamanca, Salamanca, Spain; 4 Max Planck Institute for the Structure and Dynamics of Matter, Center for Free-Electron Laser Science, Hamburg, Germany; 5 Photonics Institute, TU Wien, Vienna, Austria

**Keywords:** High-harmonic generation, Attosecond science, Quantum optics

## Abstract

High-harmonic generation underpins attosecond science. For over three decades, high-harmonic upconversion has been framed within the confines of a single-active-electron light–matter interaction featuring a well-defined cutoff photon energy. Here we demonstrate experimentally that correlated electrons can propel high-harmonic emission beyond this single-active-electron limit, markedly increasing the generated photon energies. We observe a weak secondary plateau that extends the conventional cutoff beyond 120 eV up to the water window at 280 eV. This phenomenon arises from double-electron recombination of strongly correlated electron pairs, resulting in a new cutoff scaling of up to 5.5 times the ponderomotive energy of the rescattering electrons, which notably deviates from the conventional scaling factor of 3.2. These findings reshape our fundamental understanding of the high-harmonic upconversion process and position high-harmonic generation as a potent photonic probe of attosecond-to-femtosecond electron correlations in quantum systems, opening new pathways for advanced ultrafast spectroscopy, novel attosecond source development and the exploration of strongly correlated quantum materials.

## Abstract (web summary)

The researchers experimentally demonstrate that correlated electrons can propel high-harmonic emission beyond the single-active-electron limit, markedly increasing the generated photon energies. The team observes a weak secondary plateau that extends the conventional cutoff beyond 120 eV up to the water window at 280 eV.

## Main

High-order harmonic generation (HHG) is an extreme nonlinear optical process that enables the generation of coherent extreme ultraviolet (EUV) and X-ray light similar to that of conventional lasers^1–4^. Since its discovery, HHG has played a pivotal role in advancing the understanding of light–matter interactions at unprecedented attosecond timescales and has found widespread applications in various fields, including ultrafast spectroscopy, multidimensional imaging and attosecond science^5–12^. The tabletop EUV–X-ray sources have reached a new level of performance, delivering coherent light with high brightness, excellent spatial coherence and attosecond-scale temporal structure^13,14^. Coherent X-rays have the ability to penetrate thick, opaque objects, visualize subwavelength nanostructures, control spin and magnetic currents, and access materials’ chemistry and structure at nanometre-to-picometre length and femtosecond-to-attosecond temporal scales^15,16^, potentially breaking the zeptosecond barrier. The quantum nature of the high-harmonic process was established in the 1990s^17^, and extensive research has focused on elucidating the underlying mechanisms and optimizing the upconversion efficiency from laser light to X-ray harmonics^14,18,19^.

In the past decades, theoretical investigations have also suggested that multi-electron effects and electron–electron correlation can substantially impact the structure of the high-harmonic spectrum using infrared–visible–ultraviolet (UV) driving lasers^20–26^. Electron correlation refers to quantum interactions between electrons in an atom, molecule or solid, resulting in intricate electron dynamics influenced by the presence of other electrons. As a quantum three-body system, the helium atom serves as an ideal platform for probing the strongest electron-correlated dynamics, owing to its simple electronic structure comprising only two electrons bound to a nucleus. The absence of inner-shell electrons eliminates screening effects, allowing direct and well-resolved electron–electron interactions. When interacting with a laser field, the pronounced electron–electron correlation effect in helium atoms was demonstrated to play a pivotal role in the deeply studied double ionization mechanism^27–33^. While the role of electron correlations has been extensively studied in various contexts^12^, its influence on the HHG process has received insufficient attention in experiments, partly due to the difficulties in identifying the signatures of collective electron dynamics in the high-harmonic spectrum, which is typically dominated by effective single-electron quantum pathways.

In this study, we present experimental observations of a secondary plateau in the high-harmonic spectrum, extending into the X-ray region up to the water window using a UV driving field with photon energy of 3.1 eV. The extended weak secondary plateau is attributed to the finite probability of double recombination of highly correlated electrons in gas-phase helium atoms^28,34–37^, where the collective correlated pathway of two electrons acquiring high kinetic energy and recombining to the parent ion enables the emission of a photon with energy much higher than that in conventional single-electron recombination (SER). By employing a UV laser system and a gas-filled hollow-core waveguide, we conducted comprehensive studies to explore the characteristics and origins of this extended secondary plateau, which essentially is a signature—a spectroscopic sensor—of strong electron correlations. Notably, intense EUV, UV and visible lasers are particularly suitable for bright harmonic generation because they combine favourable single-atom quantum and macroscopic phase-matching physics. Generating high harmonics with short-wavelength lasers benefits from high conversion efficiency, ultranarrow bandwidth harmonics with a large energy separation, extended X-ray cutoffs from ions, inherently naturally compressed attosecond pulse structures, effective phase and group velocity matching, increased tolerance to phase mismatch, and excellent spatiotemporal coherence^14,38^.

## Experimental set-up and observations

**Fig. 1:**
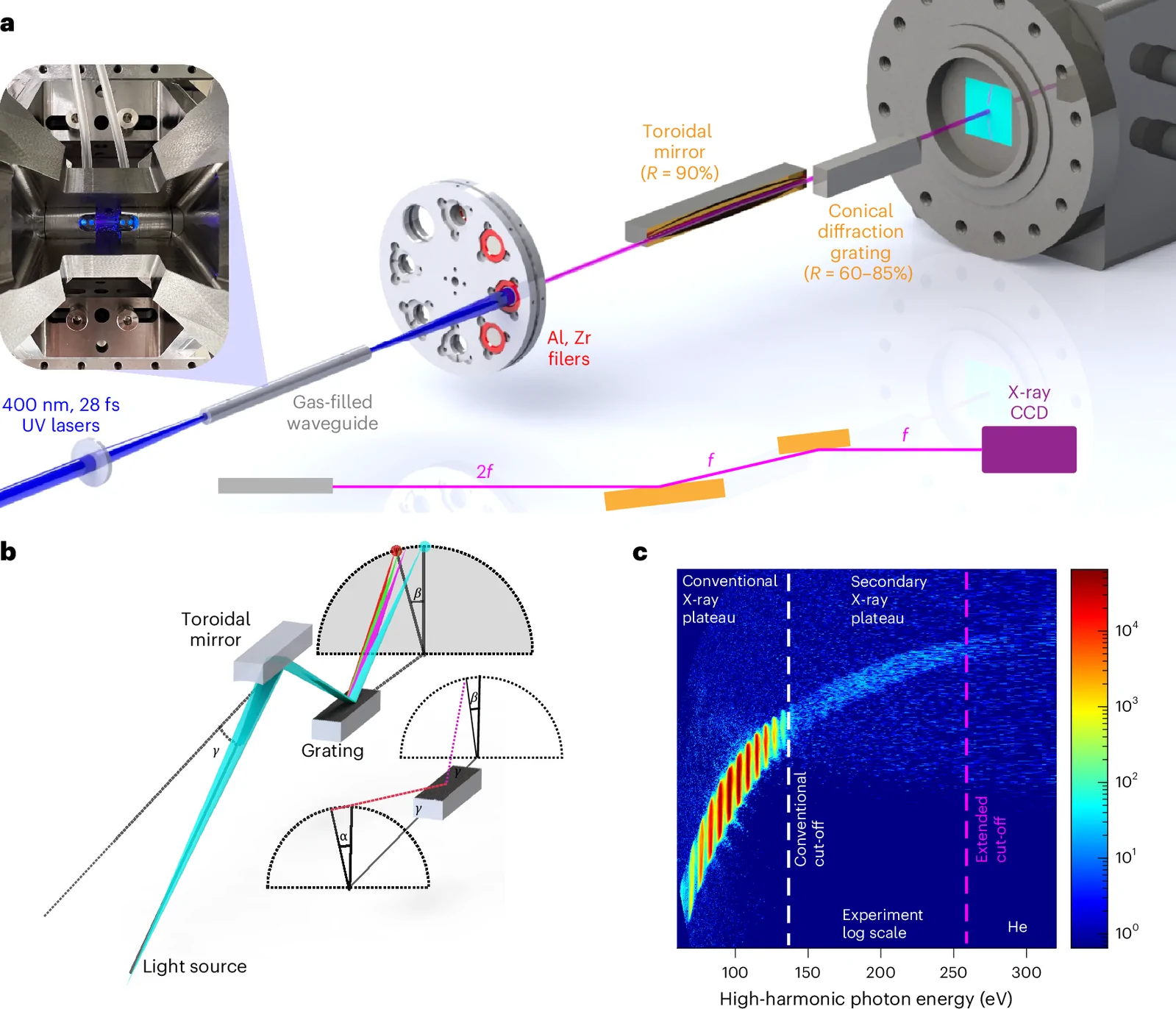
Electron correlation spectroscopy based on UV-driven high-harmonic generation in the soft X-ray regime using highly efficient conical diffraction detection. **a**, The bright high-harmonic generation is driven by 400-nm UV laser pulses coupled into a gas-filled hollow-core waveguide. The coherent X-ray emission is analysed via a conical diffraction spectrometer with a dual-optic architecture with an overall 60–85% efficiency. The diffracted signals are collected by a charge-coupled device (CCD) camera. **b**, Schematic of the highly efficient conical diffraction instrument consisting of a toroidal mirror and a plane conical grating. **c**, Experimental conical diffraction image on a photon energy scale of the high-harmonic emission from He. The horizontal projection of the conical diffraction directly represents the photon energy on a linear scale. The dashed vertical lines indicate two observable cutoffs in the experimentally retrieved high-harmonic spectrum. Extended secondary cutoffs are observed only in He and up to the water window. Source data

We perform our experiments using a 400-nm UV laser with a pulse duration of 28 fs and energies up to 2.8 mJ at 1 kHz. The UV laser light was generated through the second-harmonic upconversion of a Ti:Sapphire laser amplifier. The high harmonics are generated by coupling the UV laser pulses into a gas-filled hollow-core waveguide. The emitted radiation was collected and analysed using a highly efficient dual-optic conical-diffraction X-ray spectrometer^39,40^ (Fig. 1a,b).

**Fig. 2:**
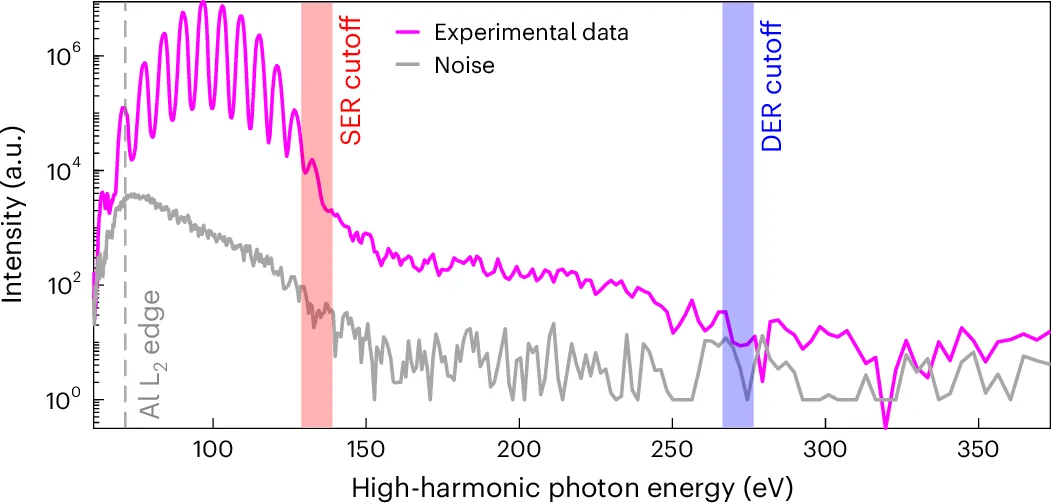
Experimental high-harmonic spectrum illustrating SER and DER signals. The spectrum shows a conventional SER cutoff (shaded red area) around 120–140 eV, and the spectroscopic signature of DER of strongly correlated electrons extending to a DER cutoff (blue-shaded area) around 280 eV in the soft X-rays. The spectral region between the two cutoffs corresponds to the secondary high-harmonic plateau—a spectroscopic sensor of electron correlation. Source data

In our experiments, we observed bright emission extending up to 120–150 eV from helium atoms (He) and argon atoms and ions (Ar, Ar^+^ and Ar^2+^), within the effective phase-matching conventional cutoffs for UV driving lasers. We have successfully reached the soft X-ray spectral region using a 400-nm driving laser. Interestingly, at laser intensities greater than 2 × 10^15^ W cm^−^^2^, we observed a distinctive feature starting to emerge in the helium spectrum—a secondary plateau extending well beyond the conventional cutoff (Figs. 1c and 2). The emission is detectable up to 280 eV owing to the combined optimization of the efficiency of the high-harmonic process and the ultralow noise floor in the detection system (Fig. 2). The extended secondary plateau is absent in the high-harmonic spectroscopic measurements in argon and neon, highlighting the unique electron correlation effect in helium.

## Double recombination and electron correlation dynamics

**Fig. 3:**
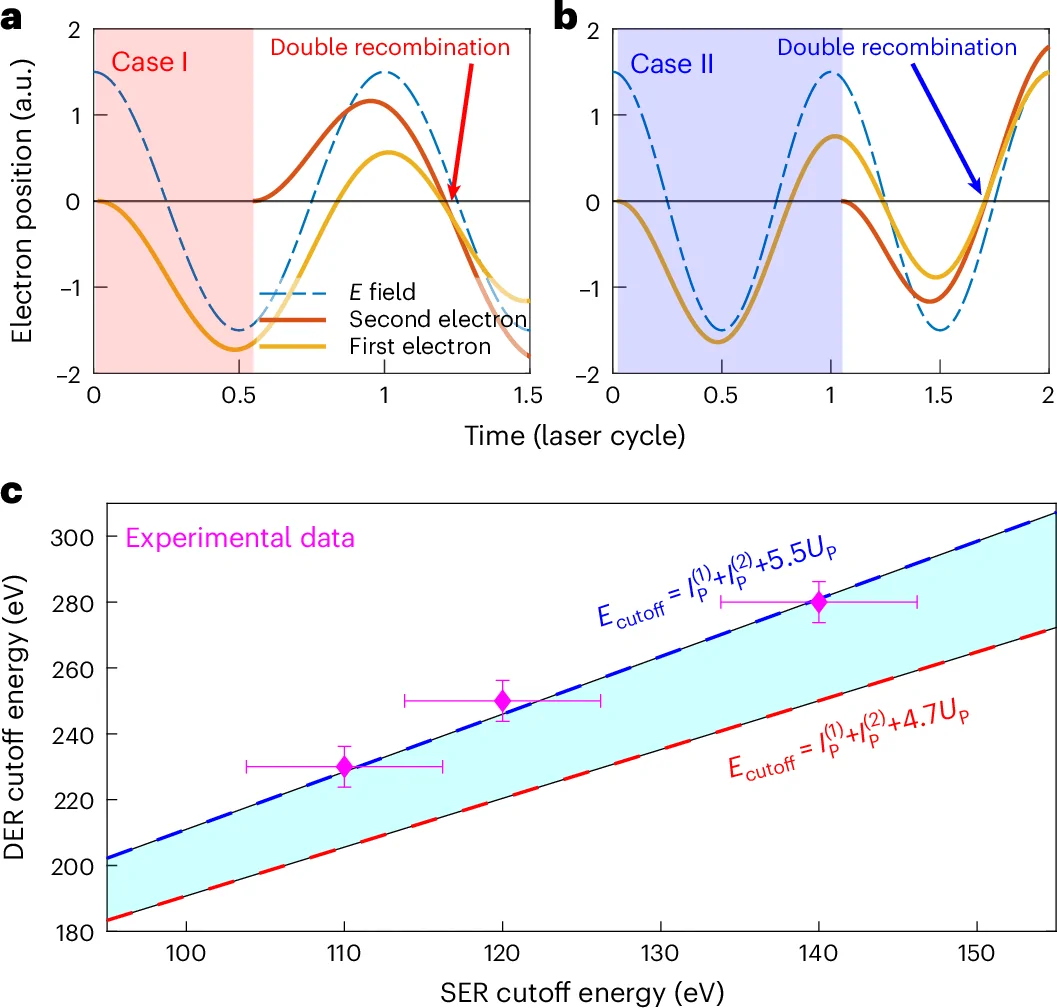
Extended high-harmonic cutoffs and cutoff rules in DER. **a**,**b**, Classical trajectory analysis: ionization of two electrons happens within an approximately adjacent half-driving laser cycle (**a**); ionization of two electrons occurs within a full adjacent driving-laser cycle (**b**). The solid yellow and red lines represent the one-dimensional electron trajectories of the first and second laser-ionized electrons, while the dotted blue line represents the laser electric field. The shaded red and dark-blue areas represent the temporal delay between the ionization of the two correlated electrons. **c**, Linear relationship between the DER and SER cutoffs indicating higher ponderomotive energies of the rescattering electrons in DER with 4.7*U*_p_ or 5.5*U*_p_ scaling. Experimental data points—plotted at (110, 230), (120, 250) and (140, 280)—include ±6.2 eV error bars corresponding to the high-harmonic spacing. Source data

To understand the origin of the unexpected extended secondary plateau, we investigated the possible mechanisms that could extend the high-harmonic spectra to higher photon energies at the single-atom level. Time-dependent Schrödinger equation (TDSE) calculations along the laser polarization direction^23^ indicate that double-electron recombination (DER) is the most likely physical process responsible for the secondary plateau, as it is the only theoretically available mechanism capable of emitting high-order harmonics at such a high photon energy range. The high-harmonic emission from double recombination requires the two electrons to be correlated^28,34–37^, enabling photon emission from their synchronous recombination. In this process, fractions of the two-electron wavepackets, entangled via non-local causality, are then ionized at different half or full-driving laser cycles and follow distinct quantum paths, mitigating strong repulsion during local space-time correlation. Afterwards, these two electrons recombine with the parent ion simultaneously, resulting in the emission of a single photon with extremely high photon energy in the soft X-ray regime. The classical trajectory analysis in Fig. 3a,b shows the typical DER trajectories of two electrons. The first ionized electron will be in a ‘long trajectory of high order rescattering’ mode so that it can re-encounter the original ion position more than once (a high-order rescattering). The second ionized electron will be in a ‘short trajectory of a single rescattering’ mode with only one possible rescattering event in the recombination^41^. Note that the terminology of ‘short’ and ‘long’ trajectories here refers to different electrons, which constitutes a major departure from the standard single-electron trajectories in HHG. The two-electron pathways require a longer phase-matching window and exhibit more complex quantum diffusion than that of a single-electron wavepacket scenario. A critical issue is that the double-electron trajectories are associated with high kinetic energies showing a different scaling with the ponderomotive energy (*U*_p_): 4.7*U*_p_ is expected for the second-order rescattering depicted in Fig. 3a and Feynman diagram in Supplementary Fig. 6b, and 5.5*U*_p_ for the third-order rescattering in Fig. 3b and Feynman diagram in Supplementary Fig. 6c, instead of the conventional scaling of 3.2*U*_p_ (refs. ^34,35^). This allows a substantial extension of the photon energy cutoff in DER. The extended secondary cutoff retrieved in the experiment matches the scaling of the DER cutoff (Fig. 3c. By varying the driving laser intensity, we observed that the DER cutoff energies scaled as 5.5*U*_p_, consistent with theoretical semi-classical calculations. The DER cutoff is defined as the point at which the weakly extended signal approaches the same magnitude as the background noise level. For example, in Fig. 2, the DER cutoff is identified at approximately 270–280 eV.

**Fig. 4:**
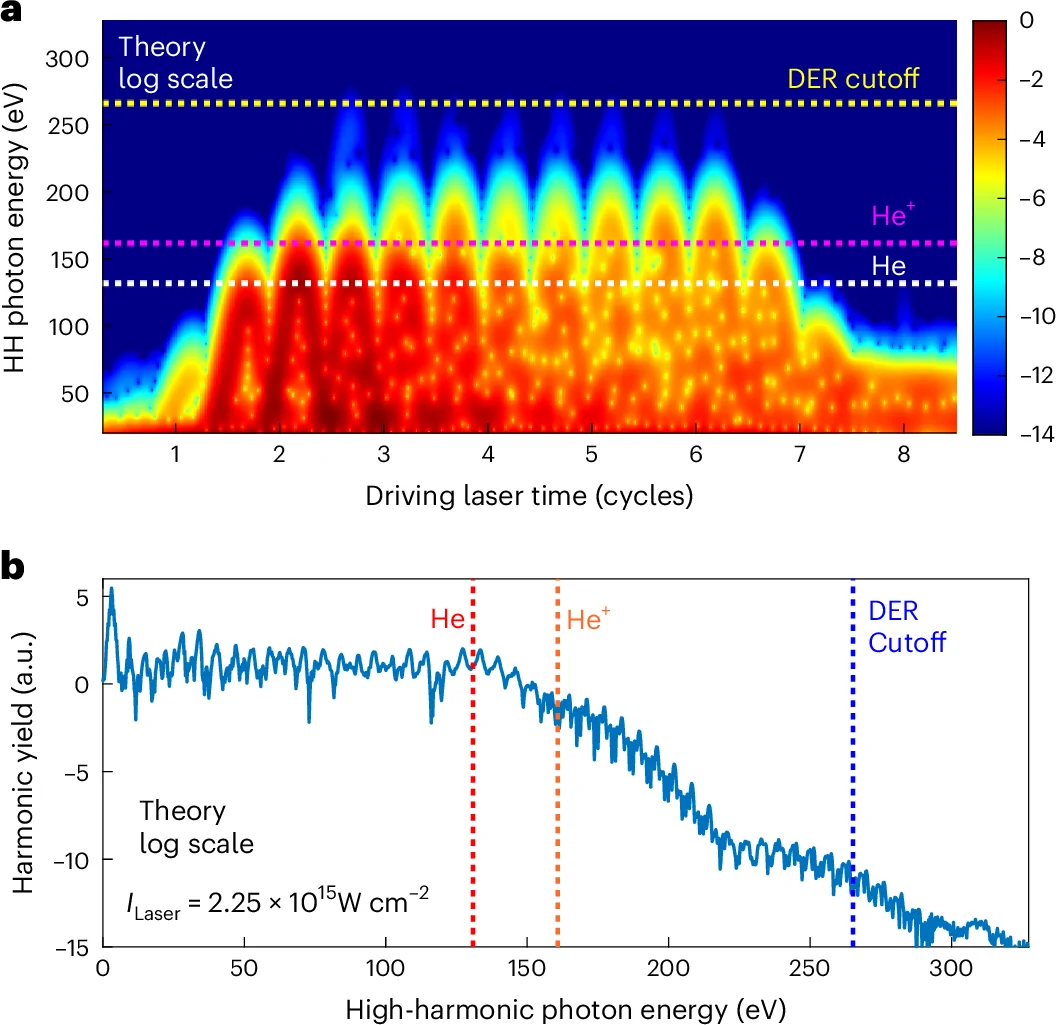
Single-atom TDSE calculations illustrating a weak DER plateau. **a**, Time–frequency analysis of the high-harmonic (HH) emission depicting three plateau regions for He, He^+^ and DER. **b**, Theoretical high-harmonic spectrum for a single He atom with a peak driving laser intensity of 2.25 × 10^15^ W cm^−^^2^. Source data

The full TDSE analysis along the laser polarization direction is also consistent with the experimental data in terms of harmonics from SER and DER cutoff photon energy (Fig. 4). Calculations are performed for a driving laser pulse of 2.25 × 10^15 ^W cm^−^^2^ peak intensity, 400 nm central wavelength and eight full-duration cycles, considering a standard sinusoidal squared envelope. The methodology follows ref. ^23^, and the numerical mesh is chosen with a full length of 1,200 a.u., and spatial and temporal steps of d*x* = 0.20 a.u., d*t* = 0.11 a.u. to guarantee convergence. The time–frequency analysis in Fig. 4a shows the photon energy emission during the laser pulse. The DER emission in the form of a weak extended secondary plateau emerges only at 2.5 cycles and later, indicating the long duration of the process (more than a laser cycle) and the requirement of a high amplitude of the external electric field. The high-harmonic spectrum depicted in Fig. 4b shows three cutoffs corresponding to SER in He and He^+^ and DER. The computed spectral range in photon energy is consistent with the experimental data. However, the three-plateau yield ratio differs substantially from the experimental high-harmonic spectrum (Fig. 2). The yield ratio between the observed SER and DER plateaus is close to four orders of magnitude versus the nearly ten orders of magnitude present in the theoretical single-atom calculations. At the single-atom level, the DER HHG mechanism involving sequential ionization of two electrons and double recombination is considerably more restrictive than conventional single-electron dynamics, resulting in a markedly lower conversion efficiency. It constitutes a higher-order process where electron–electron correlation plays a crucial role in the cross-sections of these processes. Furthermore, as indicated in Fig. 3a,b, DER requires an additional relative phase constraint between the two-electron pathways for synchronous recombination, and the extended two-electron excursion time inherently leads to greater quantum diffusion of the wavepacket.

The stronger-than-expected signal of the secondary plateau from DER can probably be attributed to two factors. First, the high-harmonic generation was intentionally overdriven, resulting in more plasma induced by the driving laser than under good phase-matching conditions. This overdriving weakens the conventional plateau but accelerates the ionization of additional electrons, thereby enhancing potential multi-electron effects. Second, macroscopic phase-matching effects, which are computationally expensive to simulate for this process (requiring thousands or millions of long single-atom calculations), play a crucial role in this process. The full sequence, from the ionization of the initial electron to the concurrent recombination of two electrons with the emission of a single photon, spans more than one laser cycle. Observing this weak extended region necessitates a duration exceeding one full driving laser cycle to satisfy the phase-matching condition. For a typical near-infrared driving laser, the temporal phase-matching window usually encompasses only one laser cycle^42^. This limited window makes it unlikely to observe the DER process. By contrast, for a UV driving laser, the high refractive indices of atoms and ions enable more laser-induced plasma build-up and a broader temporal phase-matching window, allowing multiple driving laser cycles to be phase-matched^13^. Moreover, when using a UV driving laser, the limiting critical ionization level of the gas for effective phase matching is much higher than for conventional phase matching using infrared drivers, thus greatly encouraging multi-electron dynamics to take place.

## Ellipticity dependence of SER and DER high-harmonic plateaus

**Fig. 5:**
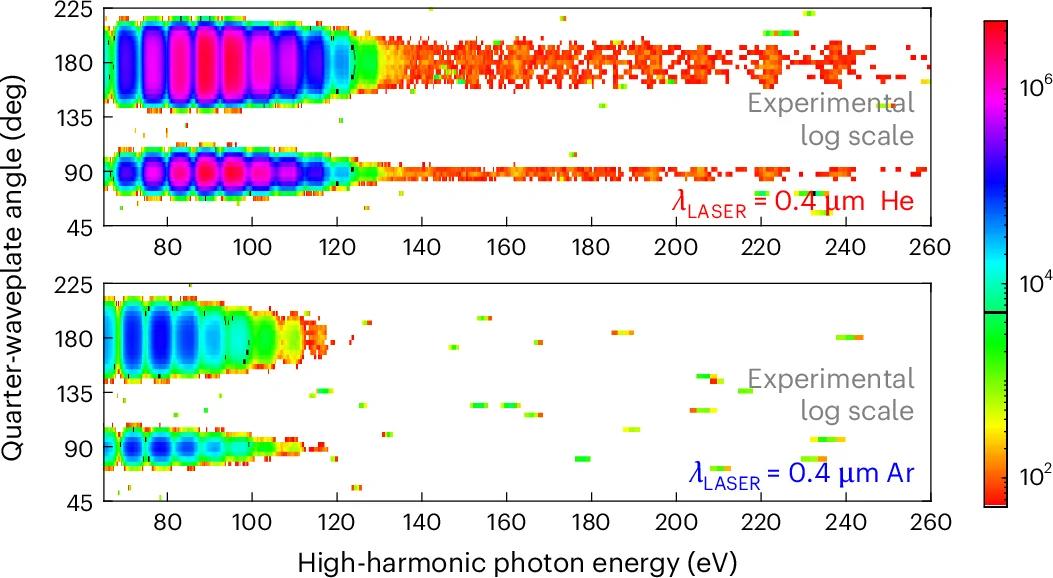
Signatures of attosecond strong electron correlations depending on the ellipticity of the UV driving laser field. Top: observation of an extended DER cutoff ranging from approximately 130 to 260 eV in helium driven by a 400-nm UV laser. Bottom: ellipticity scan using argon gas showing no evidence of an extended DER cutoff, consistent with weaker electron correlation effects. Driving pulse polarization relative to the Brewster window (p-pol, parallel-polarized: 180°; s-pol, perpendicular-polarized: 90° quarter-waveplate angle) broadens the ellipticity tolerance for HHG. Source data

To confirm the coherence of the rescattering process and exclude the possibility of plasma radiation, we conducted experiments varying the driving laser ellipticity with different gases (Fig. 5). The conventional SER plateau is present for all gas tested and shows a strong dependence on the ellipticity of the driving laser. As is well known in the HHG community, this arises from the low probability of rescattering associated with high ellipticities^43^. Both simulations and experiments confirm that no high-harmonic generation is observed under circularly polarized driving lasers, in either gas or solid phase.

By adding a certain ellipticity to the laser pulse, the electron motion acquires an off-axis component, following more intricate trajectories. A remarkable feature is that the high-photon-energy extended plateau is only observable for He atoms, suggesting that the unique strong correlation in He favours the DER process compared with other atomic species. Even with a 30-min exposure using argon gas, no extended signal was detected. By contrast, with helium, the extended signal becomes discernible within just a few seconds of exposure. The comparison spectrum is presented in Supplementary Fig. 4. The data further indicate that the secondary plateau exhibits a noticeable dependence on the ellipticity of the driving laser, as detailed in Supplementary Fig. 6. In DER, two correlated electron trajectories must simultaneously rescatter, which is less probable for off-axis trajectories. The increased ellipticity sensitivity for longer rescattering times and higher-order trajectories suggests a distinct ellipticity dependence for this process. Nonetheless, confirmation of this observation necessitates quantum mechanical simulations of double wavepacket dynamics within the ionic continuum. In Fig. 5, asymmetries are observed between the signals driven by lasers with s- and p-polarized driving lasers. These are likely attributed to the Brewster window in the beamline and the aberrations introduced by both the toroidal mirror and the grating, both of which operate at very small grazing incidence angles of 2°.

Importantly, both the conventional and secondary plateaus vanish at large ellipticities of the driving laser, confirming the rescattering nature of the DER process. This implies that DER harmonics are coherent, as expected from the rescattering mechanism.

## Possible mechanism

After evaluating several potential mechanisms, the DER process appears to provide the most consistent explanation for our observations. In this work, the emergence of a new, extended cutoff—or secondary plateau—occurs only when the HHG process is overdriven with high laser intensity. At lower driving intensities, although phase-matching and coupling conditions are more favourable and the high-harmonic flux can increase by more than two orders of magnitude, no sign of cutoff extension is observed. One potential mechanism that could account for such an extension is high-harmonic wave mixing. While infrared–EUV wave mixing has been previously reported^44^, the degree of cutoff extension observed in those studies is vastly smaller than in our findings. Wave mixing between EUV photons has also been speculated, but it has not been experimentally demonstrated, and it is typically expected to occur under conditions of optimized HHG flux, rather than in the overdriven regime observed here.

Second, our experimental results show clear evidence of high-harmonic emission from multiply charged argon species; here, Ammosov–Delone–Krainov (ADK) and TDSE calculations show that emission from Ar^2+^ is present. In this case, the cutoff extension manifests as a shift of the main plateau to higher photon energies, contrasting with the weak extensions observed in helium. A similar mechanism involving He^+^ could also contribute; however, high-harmonic emission from He^+^ has not been definitively reported so far. Although He^+^ can undergo ionization under specific conditions, our experimental parameters are well outside the tunnel ionization regime, such that efficient high-harmonic generation from He^+^ is not expected. Even so, He^+^ has an ionization potential 29.8 eV higher than neutral He (${I}_{P}^{(1)}$ = 24.6 eV and ${I}_{P}^{(2)}$ = 54.4 eV), implying that the expected cutoff extension should be on the order of 30 eV, which is well below the over 100 eV extension we observe.

Third, the new cutoff energy is consistent with the DER cutoff predicted by theory. In our system, reabsorption does not dominate the HHG spectrum in the soft X-ray region covered by our observations, and the conventional cutoffs for argon and helium align well with the well-known cutoff law. The observed scaling of the secondary cutoff with approximately 5.5 times the ponderomotive energy is quantitatively consistent with DER and strongly indicative of a different underlying quantum physics mechanism.

Taken together, these observations support the interpretation that a correlation-driven double-electron process is the most plausible explanation for the extended high-harmonic cutoff observed in our experiments as a distinct, weak, secondary plateau spanning up to 140 eV beyond the conventional cutoff.

## Conclusions and outlook

In summary, we experimentally observed an extended secondary plateau in the UV-driven high-harmonic spectrum of helium, reaching the water window of the soft X-ray region. Absent in argon under comparable conditions, this feature arises from the double recombination of correlated electron pairs and provides direct evidence of electron-correlation effects in high-harmonic generation. The plateau extends to cutoff energies up to 5.5 times the ponderomotive energy, far beyond the conventional single-electron limit of 3.2. Numerical simulations identify a DER process involving ionization across multiple laser cycles and coherent rescattering as the origin of the extended emission. The process is enhanced by reduced quantum diffusion under short-wavelength driving and by effective phase matching over multiple half cycles, enabling the macroscopic build-up of higher-order rescattering contributions. Ellipticity-dependent measurements further confirm its coherent nature.

Multiple plateau structures have also been observed in high harmonics generated from liquid-phase targets, attributed to high-order scattering and off-site recombination, indicating that similar mechanisms may also arise in amorphous condensed-matter systems^45–47^. Secondary plateaus in solid-state HHG have been described within single-particle Bloch-electron dynamics involving excitation to higher conduction bands^48–51^. Beyond this picture, correlated-electron recombination in Mott insulators has been predicted to generate HHG through doublon–holon annihilation^52^, where the plateau spectral range reflects the distribution of doublon–holon pair energies in the presence of the driving field. This mechanism bears a conceptual resemblance to the two-electron recombination process described here for atoms, suggesting that many-body correlation effects may provide a unifying route to plateau extension across atomic and condensed-matter HHG systems^53^. More broadly, the deviation from the semi-classical three-step model highlights the need for a quantum description of correlated-electron dynamics within the strong-field approximation^54^. The secondary plateau thus emerges as a promising platform for probing femtosecond-to-attosecond electron–electron dynamics and exploring local and non-local correlations in complex quantum systems^55^.

Looking ahead, attosecond pump–probe investigations of the DER process could potentially resolve the temporal evolution of electron correlations during recombination. Extending correlation-sensitive HHG spectroscopy to solids^46,56^, liquids^45^ and complex molecules may uncover tailored electronic and optical properties for ultrafast photonics and quantum materials. Although the extended X-ray emission remains intrinsically weak due to low densities of correlated electron pairs and stringent recombination conditions, engineering media with enhanced correlated electron-pair abundance or interelectron correlations could yield brighter DER HHG signals, enhancing high-harmonic sources for advanced spectroscopy and ultrafast dynamics studies. The high-harmonic yield may also serve as a spectroscopic probe of electron-pair entanglement, with implications for correlated quantum materials and attosecond sensing.

## Online content

Any methods, additional references, Nature Portfolio reporting summaries, source data, extended data, supplementary information, acknowledgements, peer review information; details of author contributions and competing interests; and statements of data and code availability are available at https://doi.org/10.1038/s41566-026-01976-2.

## Supplementary information


Supplementary InformationSupplementary Sections 1–8, including Supplementary Figs. 1–10.
Peer Review File


## Source data


Source Data Figs. 1–5.Source Data Fig. 1: 2D intensity matrix of the spectrum image for Fig. 1c. Source Data Fig. 2: Plotted experimental high-harmonic spectrum data. Source Data Fig. 3: Calculated electron trajectories and E-field (Figs. 3a,b), and experimental/theoretical comparison curves of DER and SER (Fig. 3c). Source Data Fig. 4: 2D surface plot data for the time–frequency analysis (Fig. 4a) and the calculated theoretical spectrum data (Fig. 4b). Source Data Fig. 5: Tabulated ellipticity scan measurement matrices for helium (upper panel) and argon (lower panel).


## Data Availability

The data displayed in Figs. 1–5 are available via Zenodo at https://doi.org/10.5281/zenodo.20788561 (ref. ^57^). Source data are provided with this paper.

## Code availability

The TDSE code is held by A.H. and is available upon reasonable request. All other computer codes used in this study are available from the corresponding authors upon reasonable request.
