# Supplementary material for: Correlated electrons extend X-ray high-harmonic generation beyond the single-electron limit
Source: Nat Photonics. 2026 Aug 7;20(9):1092–8. doi: 10.1038/s41566-026-01976-2 (PMC13533847; doi:10.1038/s41566-026-01976-2)
Supplement: Supplementary file 1 [file 41566_2026_1976_MOESM1_ESM.pdf]

nature photonics

Supplementary information

https://doi.org/10.1038/s41566-026-01976-2

Correlated electrons extend X-ray high-harmonic generation beyond the single-electron limit

In the format provided by the authors and unedited

Table of Contents

Supplementary Note I: Driving laser field for efficient high harmonic generation and efficient yield of electron correlation signal 2

Supplementary Note II: Behavior of UV-driven high-harmonics. 2

Supplementary Note III: UV-driving High Harmonic Spectrum for atomic and molecular systems. . . . 3

Supplementary Note IV: Ellipticity dependence of SER and DER plateaus. 4

Supplementary Note V: "AMO Feynman diagrams" of the six-step double electron recombination process. 5

Supplementary Note VI: Relationship between the single and double electron recombination cutoffs. . 6

Supplementary Note VII: Alternative mechanisms possibly responsible for the secondary plateau signal. 6

Supplementary Note VIII: Highly efficient spectrometer design for higher-order harmonics within the conventional and secondary plateau regions. 7

References 7

Supplementary Note I: Driving laser field for efficient high harmonic generation and efficient yield of electron correlation signal

The driving laser operates at a central wavelength of 400 nm with a pulse duration of 28 fs and a pulse energy of up to 2.8 mJ. The temporal characteristics of the ultrafast 400 nm pulses were characterized using self-diffraction frequency resolved optical gating (SD-FROG)[1]. Fig.S1 shows the retrieved FROG trace and temporal profile. Using multi-cycle driving pulses are essential for observing the multiple-rescattering events, fundamentally important for the double electron recombination which happens with higher probability.

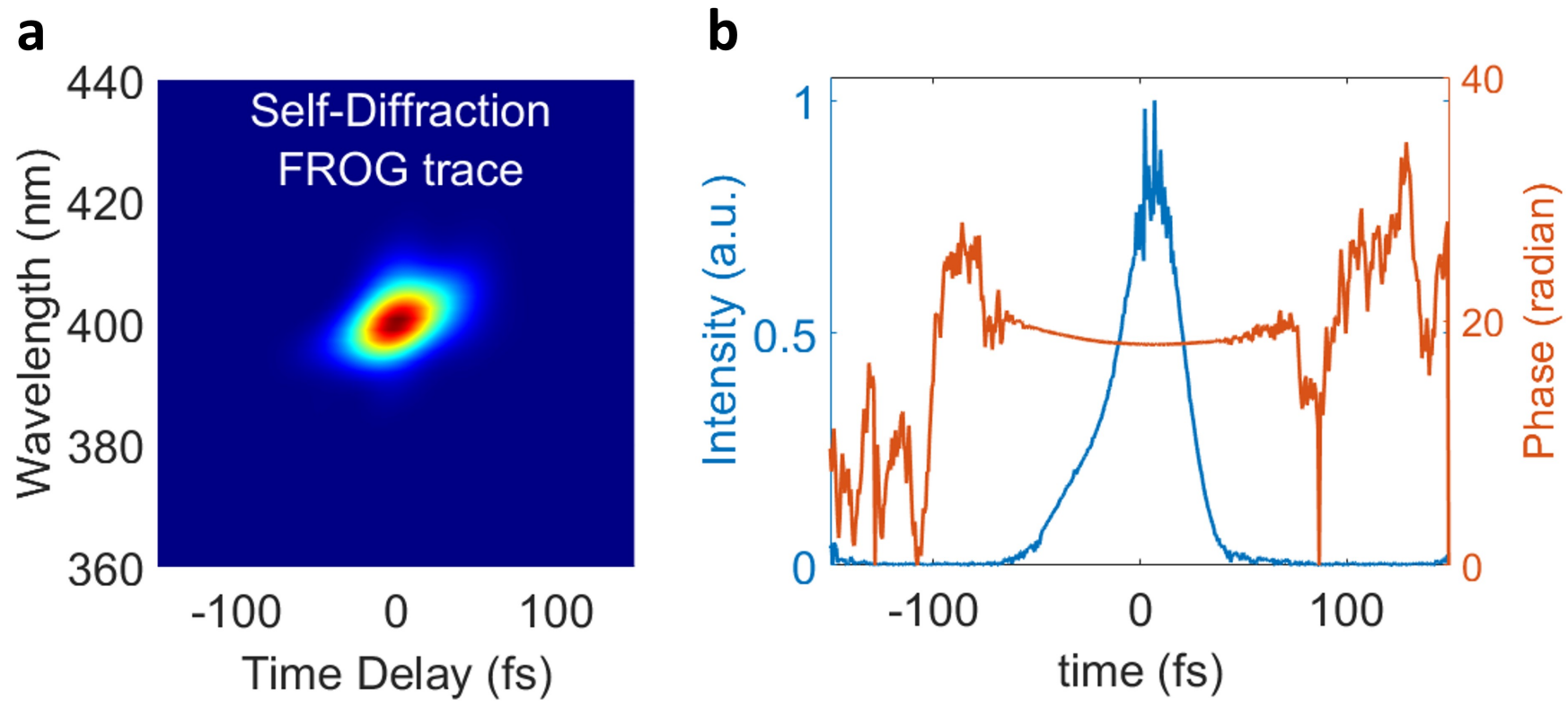

Fig. S1 Self Diffraction FROG measurement on the 400 nm driving laser. a. Experimental SD FROG trace. b. Retrieved spectral phase information, indicating a near transform-limited pulse.

Supplementary Note II: Behavior of UV-driven high-harmonics.

The UV-driven harmonic generation in a gas-filled capillary system exhibits minimal reabsorption losses in gases such as argon and neon, owing to the low phase-matching pressures required for highly efficient high-harmonic generation (HHG). Furthermore, the higher frequency of the UV driving laser (compared to conventional near-infrared drivers) enhances its penetration through plasma, enabling a longer effective interaction length[2, 3].

In our system, the observed HHG spectra align well with theoretical predictions, demonstrating that the harmonic cutoffs for all tested gases (He, Ne, At, and H2) adhere to the conventional cutoff law. Spectra from He and Argon is shown below in Fig.S2.

The beamline is designed with a minimum volume and minimum beam height to maximize overall

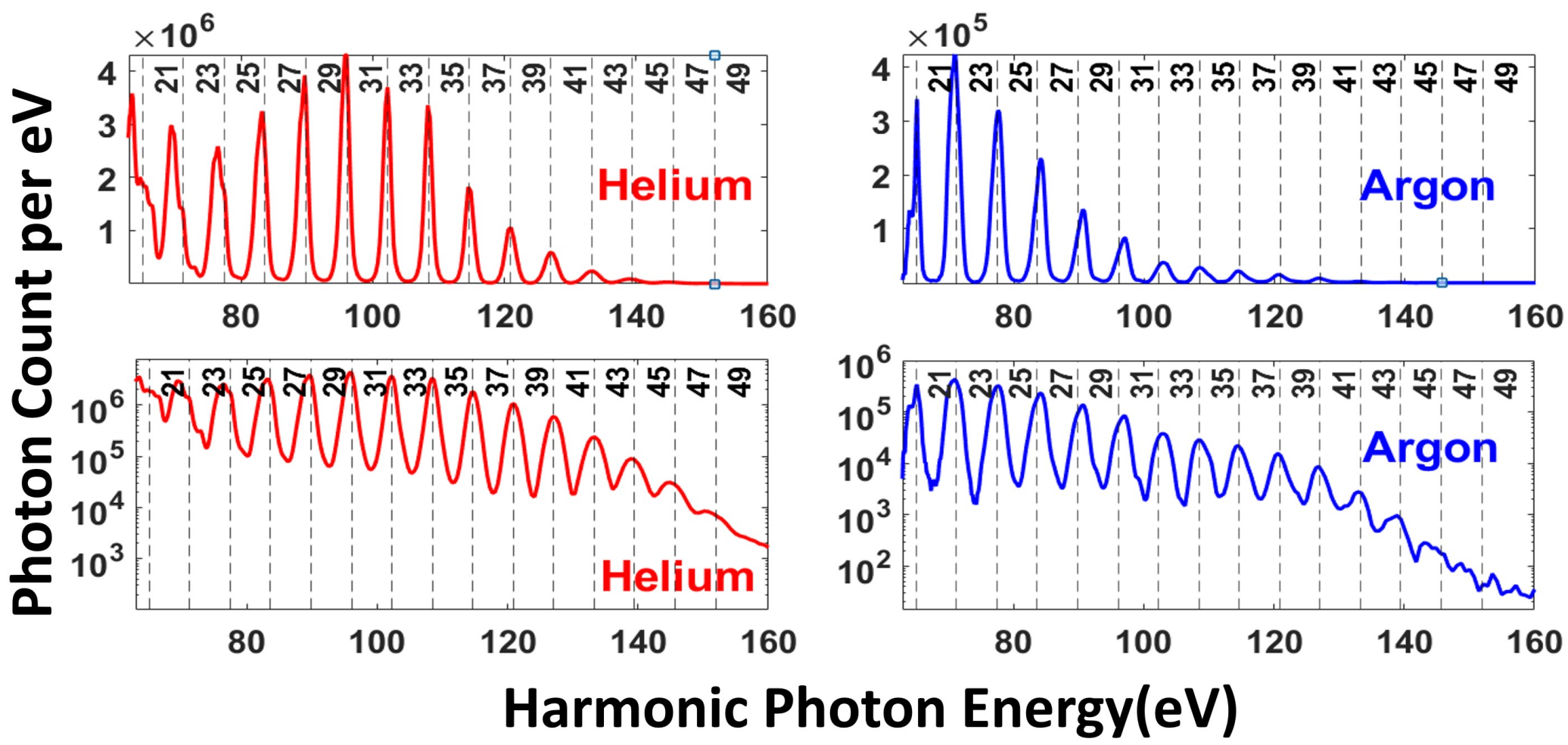

Fig. S2 Experimental high harmonic spectrum within the SER plateau region. The bright narrow-linewidth emission from He (Left) at 70-100 eV is about 1 order of magnitude stronger compared to Ar (Right). The SER cutoff for He is similar to that of Ar on logarithmic scale at the same illumination conditions at optimal pressure (Top: linear scale, and Bottom: log scale)

mechanical stability. Using specially designed modular compact chambers, we optimize vacuum to 10^{-7} - 10^{-8} torr and reduce equipment-induced vibrations. Additionally, implementing a light-tight experimental design is critical to ensure that the camera detects only the intended signal, while eliminating light from stray reflections, scattering, or clipping. These design optimizations are essential to isolate the coherent low yield signal and eliminate artifacts.

To obtain an observable double-electron recombination (DER) signal, it is essential to employ a shorter-wavelength driving laser compared to the commonly used Ti:Sapphire laser at around 800 nm. This is because the temporal phase-matching window required for the DER process is longer than that required for the single-electron recombination (SER) process. Fig.S3 shows the temporal phase-matching windows for 800 nm and 400 nm driving lasers separately, illustrating the trend of extending the phase-matching window with shorter driving wavelengths.

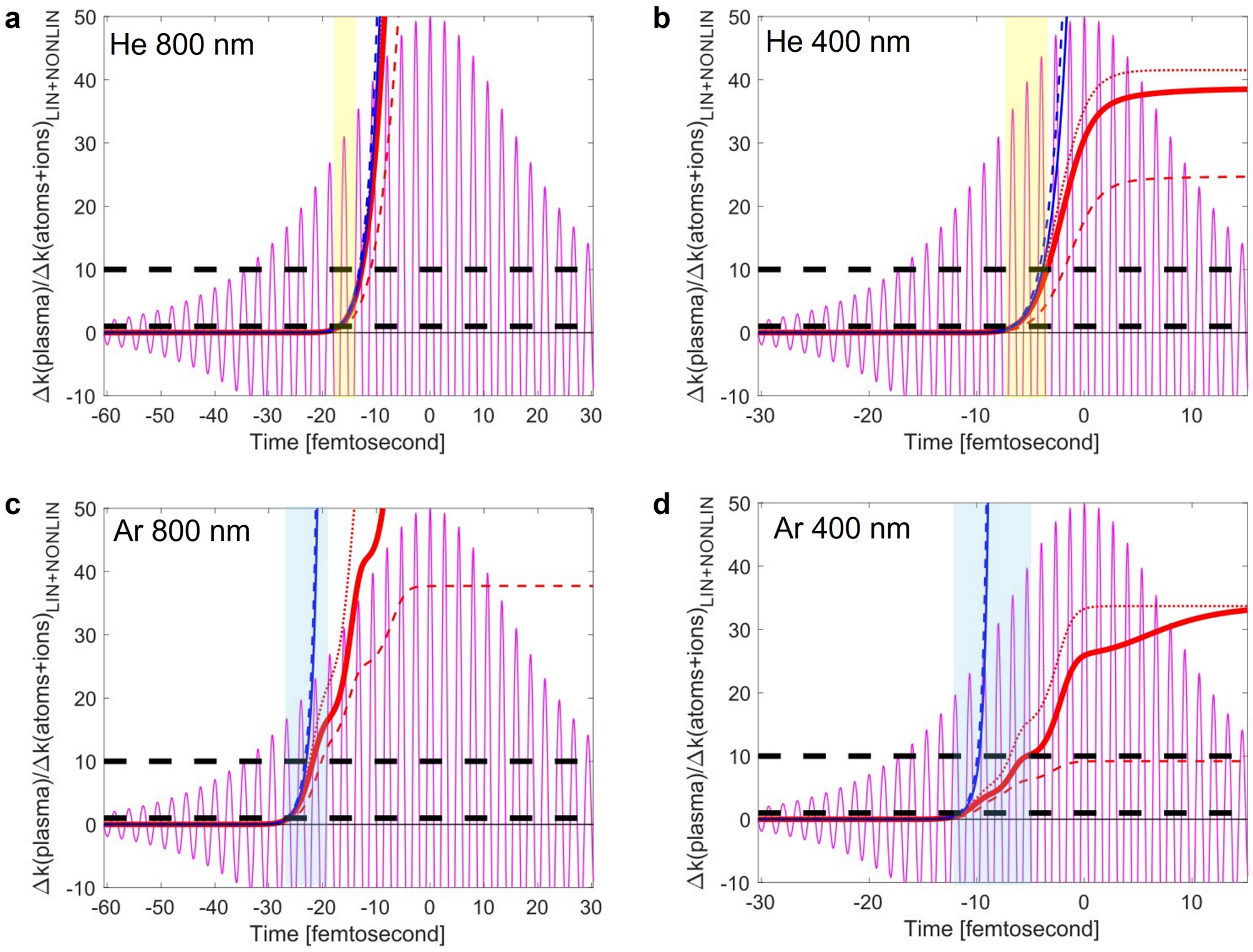

Fig. S3 Effective temporal phase-matching window comparison between 400 nm and 800 nm driving laser. Calculated ratio of the dynamic phase mismatch contributed by plasma to that of a combination of atoms and ions for helium (a, b) and argon (c, d), driven by 800 nm and 400 nm lasers, respectively. The solid blue and red lines represent the atoms-only case and the atoms-with-ions case, respectively. The shaded yellow and blue areas indicate the effective phase-matching windows. The dashed horizontal lines bracket a ratio corresponding to a coherence length of 10 microns. Same laser intensity is used for these calculation. For the helium case, the phase-matching window is approximately extended from 2 to 5 half driving laser cycles. For the argon case, the extension is from 3 to 8 half driving laser cycles.

Supplementary Note III: UV-driving High Harmonic Spectrum for atomic and molecular systems. In our experiments, we examined high-order harmonic generation using helium versus neon and argon as target gases. The harmonic spectra obtained from neon and argon exhibit similar characteristics for photon energies above the aluminum absorption edge, with neon demonstrating slightly enhanced brightness. In contrast, the spectrum generated from helium shows distinct features. The bright portion of the raw harmonic signal, as recorded by the X-ray CCD camera, is presented in Fig.S2. Additionally, as illustrated in Fig.S4, the helium spectrum exhibits a weak but measurable, reproducible signal extending beyond the conventional cutoff. This extended emission is absent in the argon spectrum, which displays a sharp and well-defined cutoff with no indication of spectral extension. The observed post-cutoff signal in helium is attributed to the double-electron recombination process, which is evidently suppressed in argon under comparable experimental conditions.

Given that electron correlation in helium can enable single-photon emission through double recombina-

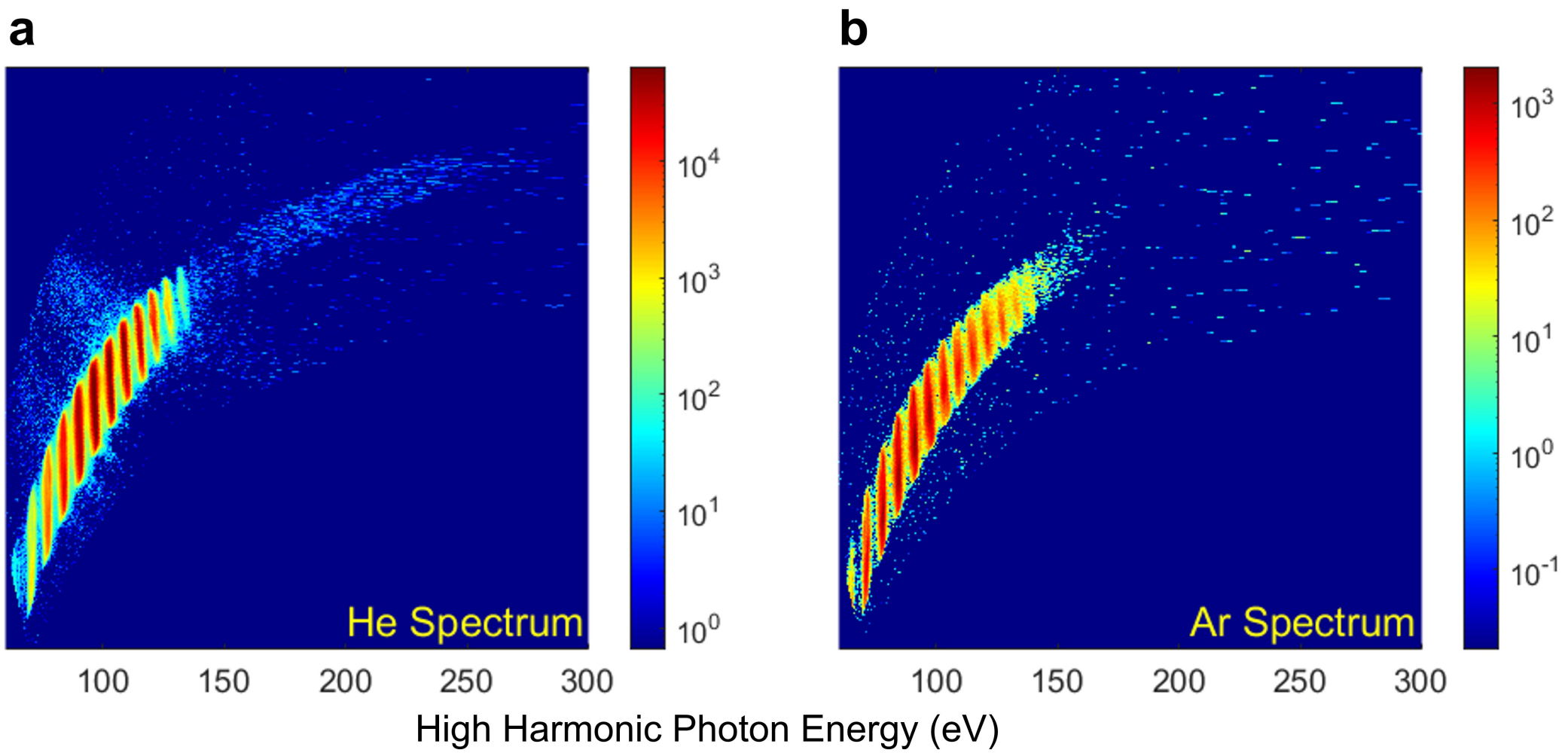

Fig. S4 Raw experimental image of high harmonic spectra on an X-ray CCD in a conical diffraction geometry. a. High harmonics from helium showing a conventional and a secondary plateau. b. High harmonics from argon gas show only a conventional plateau region under the same illuminating conditions. The yield is plotted on a logarithmic scale, illustrating approximately 4 orders of magnitude lower signal in the DER plateau compared to the SER one.

tion, we investigated whether an analogous two-electron recombination process could occur involving two electrons within a hydrogen molecule[4]. However, an anticipated signature of a secondary plateau has not been clearly resolved in our experimental observations in a parameter space analogues to that of He.

This ambiguity may stem from two potential factors: First, the electron correlations in the molecular system (H_2), with vibrational and rotational dynamics, could be significantly weaker than in helium, potentially insufficient to mediate simultaneous two-electron recombination followed by the emission of a single photon. Alternatively, the laser intensity employed in our experiments may substantially surpass the barrier suppression intensity of hydrogen[5, 6], driving the high-harmonic generation process far from optimal phase-matching conditions and thereby obscuring the expected signal. The high harmonic spectrum from hydrogen molecules is shown in Fig.S5.

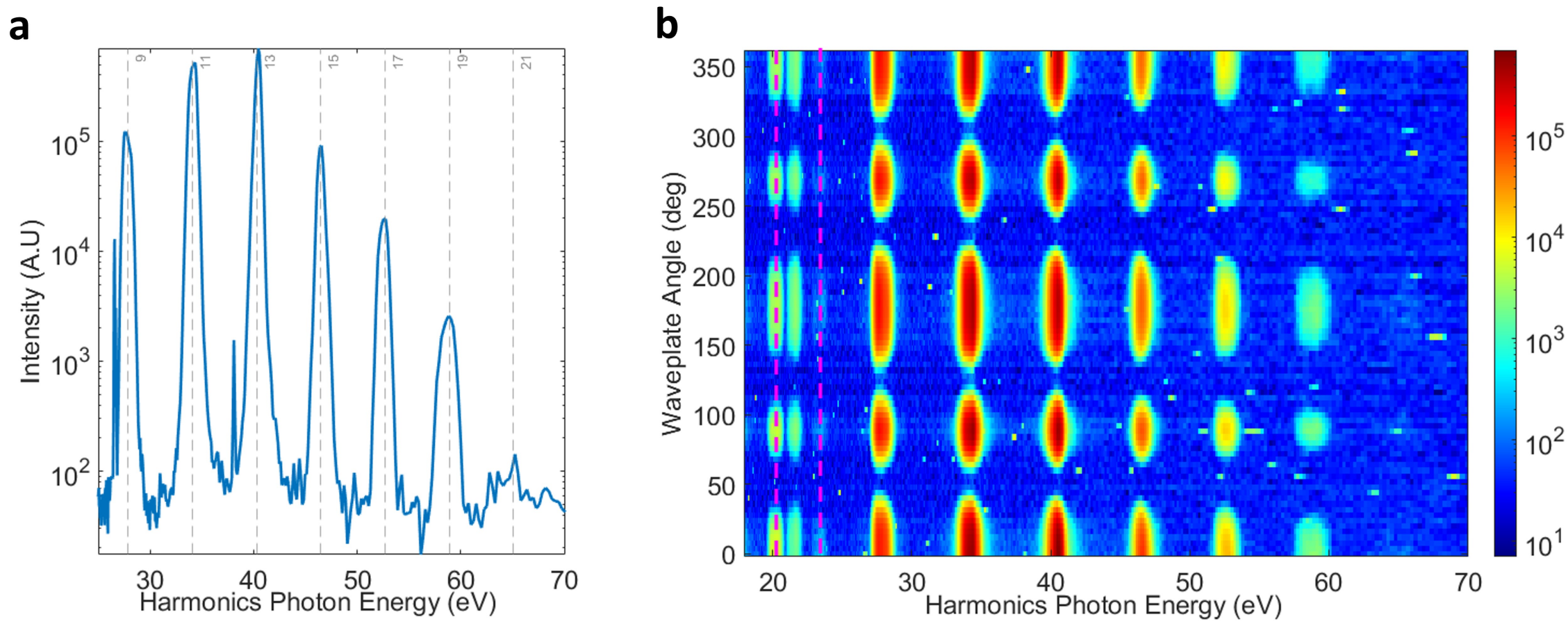

Fig. S5 Harmonic spectra form molecular hydrogen. a. High harmonic generation in hydrogen molecules. b. Laser ellipticity scan.

Supplementary Note IV: Ellipticity dependence of SER and DER plateaus.
In the DER process, the two correlated electron trajectories have to meet at a simultaneous rescattering, which renders this scenario much less probable for off-axis trajectories. Additionally, the sensitivity to the ellipticity is expected to be higher for longer rescattering times and the higher-order rescattering trajectories required in the DER mechanism.

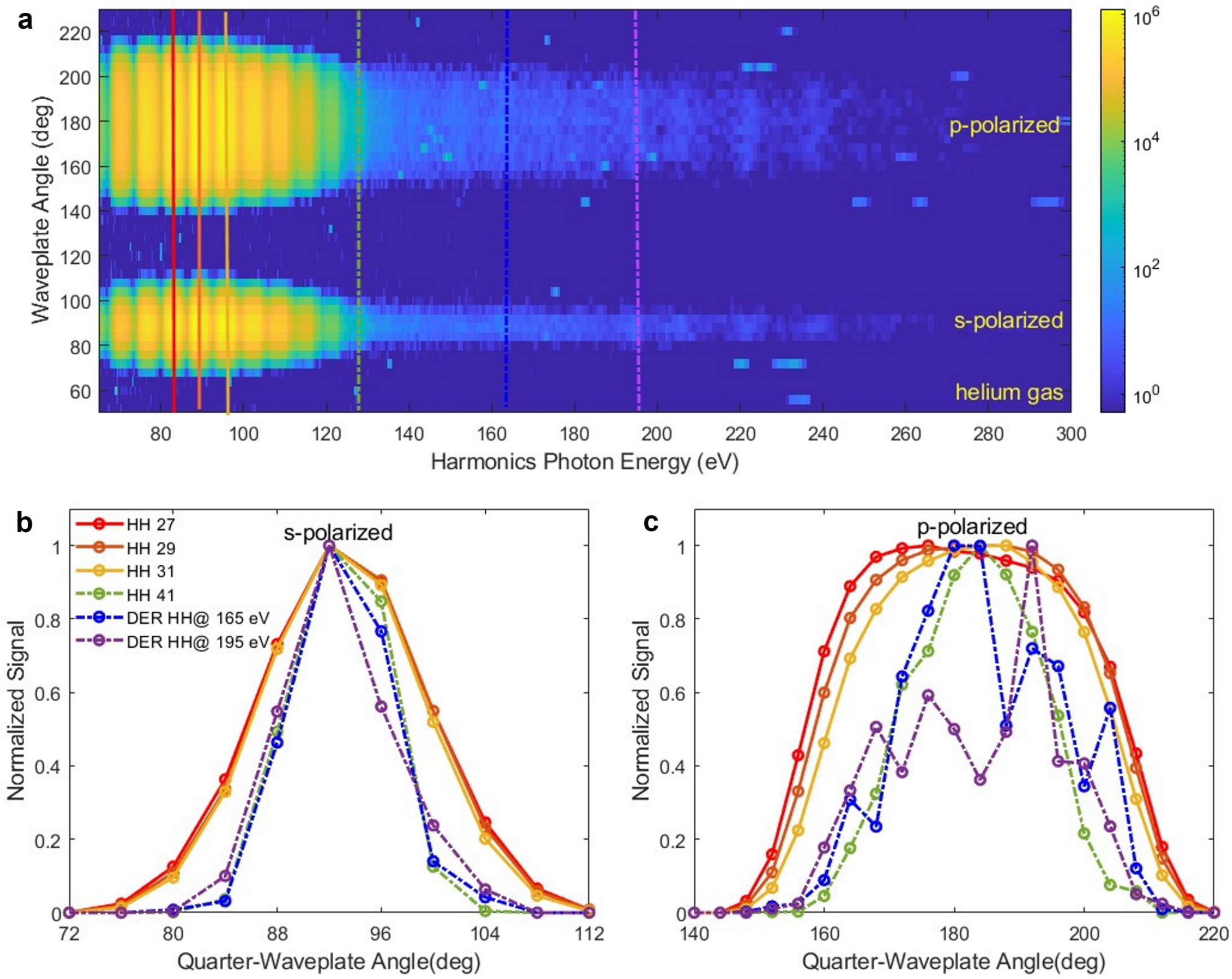

Fig. S6 a. Ellipticity scan of the driving field in helium with color-coded harmonics within the conventional and secondary plateau, selected for analysis. b. and c. Normalized selected harmonic signal centered at s-polarized and p-polarized driving laser pulses as a function of the quarter-waveplate angle, illustrating an average range of 14 degrees and 48 degrees at FWHM for s and p polarizations for the SER plateau (solid lines), and 8 and 25 degrees for the DER plateau (dashed lines), respectively.

In Fig. S6(a), we select three strong harmonics along with the last harmonics from the conventional SER plateau, and two distinct DER harmonics located around 165 eV and 195 eV. In S6(b) and (c) display the normalized signal as a function of the quarter-waveplate angle. The data indicate that the signal for the three strongest harmonics from the conventional plateau exhibits a decay at varying slopes compared to the other harmonics when the quarter-waveplate modifies the driving laser from linear polarization. Notably, the DER harmonics demonstrate a more pronounced sensitivity to changes in the ellipticity of the driving laser, which aligns with expectations. However, to achieve a comprehensive understanding and validation of these observations, quantum mechanical simulations of double wave-packet dynamics in 3D within the ionic continuum remain essential.

Supplementary Note V: "AMO Feynman diagrams" of the six-step double electron recombination process.

The process now involves two electrons. Instead of being "a doubled version" of the original three-step mechanism, an additional step arises in which the correlations of the two electrons contribute to the physics of this attosecond light-matter interaction. The Feynman diagrams are shown below in Fig.S7. The HHG in the DER plateau originates from two ionization steps, a quantum correlation step linking the ionization of two electrons, two accelerations in the continuum, followed by one simultaneous recombination step in a semi-classical picture (total 6 steps). Two mechanisms of electron correlations of bound electrons of different origins in an external laser field are depicted in the AMO diagrams - nonlocal causality and spatial nonlocality. [7, 8] The first type, the nonlocal causality or quantum entanglement, manifests itself as action-at-a-distance, where influences propagate even between non-interacting electrons, irrespective of their spatial separation (depicted with connected triangles and an interacting wave). In general, the electron correlations in this mechanism arise from interactions or symmetry constraints, in which the electrons lose their individuality. The second nonlocality, the

spatial nonlocality, occurs in the interaction between the two spatially extended electron wavepackets, or in interactions with other particles (depicted with opposing triangles and an interacting wave). This correlation is not classical and implies nonlocality in physical space-time, intimately connected to the quantum uncertainty, thereby leading to entanglement in many-body quantum systems. Both nonlocal causality and spatial nonlocality probably play a role in the DER mechanism, and the relative weight of each remains to be investigated.

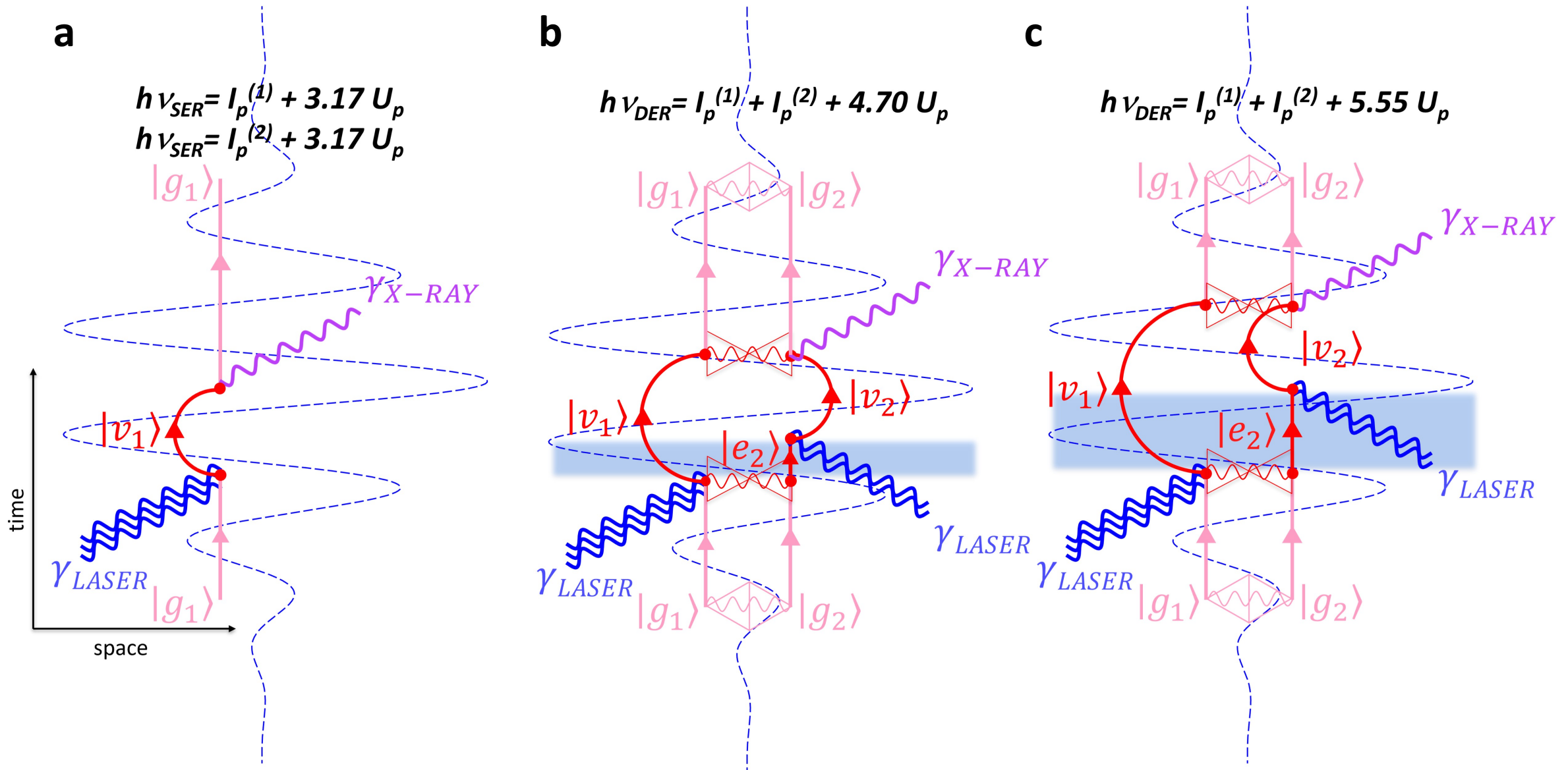

Fig. S7 "AMO Feynman diagrams". a. Feynman diagram for the conventional three-step-model of high harmonic generation. b and c. Feynman diagrams for 6-step double electron recombination model with different ionization delay between two strongly correlated electrons. The time axis is clocked by the duration of the cycle of the driving laser pulse.

Supplementary Note VI: Relationship between the single and double electron recombination cutoffs. The single electron recombination (SER) cutoff, i.e. the conventional high harmonic cutoff, has a linear relationship with the double electron recombination (DER) cutoff. For helium atoms, the DER has the following form:

$$h\nu_{DER} = A(h\nu_{SER} - I_P^{(1)}) + I_P^{(1)} + I_P^{(2)}$$

Here, $I_P^{(1)}$ and $I_P^{(2)}$ are the ionization potentials of neutral He and He+ which are 24.58738 eV and 54.41776 eV, while A is a constant that is either 1.482 or 1.75, depending on the time interval between the ionization times of the two correlated electrons (see also [9–11]). Fig.S8 presents a typical set of experimental spectral images with increasing driving pulse intensity from top to bottom. The cutoff energies for SER and DER processes are analyzed with their errors in Fig.3, indicating a linear correspondence in good agreement with the theoretical models.

Supplementary Note VII: Alternative mechanisms possibly responsible for the secondary plateau signal.
It is theoretically and experimentally compelling to investigate mechanisms that could potentially extend the high harmonic spectrum to higher photon energies. We first rule out the possibility that the observed extended HHG originates from He^+ ions within the conventional three-step model. To the best of our knowledge, no studies have reported efficient high harmonic production from He^+ ions, which ionization potential is significantly higher than that of neutral helium, requiring laser intensities well beyond the capabilities of single-stage multi-mJ Ti:Sapphire amplifiers. As shown in Fig.S9, estimates of the tunnel ionization [12] for various gases indicate that, within the experimentally accessible laser intensity range of our geometry, multiply charged ions of Ne and Ar can be produced at high ionization rates. Correspondingly, we observe experimentally efficient HHG from the resulting ions that is comparable in brightness to the yield of harmonics from the neutral atoms. In contrast, ionization of He^+ under these conditions is unlikely through conventional high harmonic upconversion due to its higher ionization threshold. The ADK model is known to overestimate the ionization level in the parameter

space of this study, hence, the actual ionization levels are lower than estimated. Furthermore, no significant pulse reshaping or pulse shortening was measured in He at the exit of the waveguide and we exclude this as is a possible mechanism of increasing the driving pulse intensity through self-confining effects in space-time. [2, 3, 13, 14]

Several theoretical mechanisms could potentially contribute to the extension of high harmonic cutoff photon energy. One of the likely candidate mechanisms that is able to extend the cutoff this far is the wave-mixing of high harmonics, a topic that has been frequently discussed in recent years but only partially speculated theoretically.

While we cannot entirely exclude the possibility that the observed signal arises from the wave-mixing of high harmonics, our observations suggest otherwise. Specifically, we did not detect any extended cutoff signal, even when achieving the highest flux of high harmonics when the phase-matched interaction length is optimized that enables near-exponential signal build-up. Also theoretically, wave-mixing should most likely result in the third harmonic of the single strongest high harmonics. However, the absence of this extended cutoff signal in our experimental setup indicates that wave-mixing is unlikely to be the primary contributing mechanism. Further theoretical and experimental investigations are necessary to fully understand the role of wave-mixing in extending high harmonic cutoff photon energy.

Supplementary Note VIII: Highly efficient spectrometer design for high order harmonics within the conventional and secondary plateau regions.
To maximize throughput efficiency, we employ a low aberration off-plane grating spectrometer design with only two optical elements. The angle of incidence of the grazing is minimized to further enhance efficiency, with both the toroidal mirror and the off-plane grating set at 2 °. The gratings used in this work to characterize the conventional and the extended secondary cutoff are with groove densities of 500 and 1200 lines/mm and with a blaze angle θ_B to be 4.7 and 8 ° respectively.
We calculated the grating efficiency for various combinations of incidence angles and groove densities, which could be applied across different harmonic wavelength ranges (see Fig.S10).
A smaller grazing incidence angle improves first-order diffraction efficiency and usually shifts its peak toward higher energies. However, excessively small angles introduce larger aberrations that may not be fully compensable. The diffraction angle follows the equation:

$$\frac{m\lambda}{d} = \sin\gamma(\sin\alpha + \sin\beta)$$

The maximum diffraction efficiency is around wavelength:

$$m\lambda_{max} = 2d\sin\theta_B \sin\gamma$$

where m is the diffraction order. The blaze angle θ_B satisfies [15, 16]:

$$2\sin\theta_B = \sin\alpha + \sin\beta$$

References

[1] Trebino, R.: Frequency-Resolved Optical Gating: The Measurement of Ultrashort Laser Pulses, pp. 117–139 (2000). https://doi.org/10.1007/978-1-4615-1181-6_6

[2] Popmintchev, D., Hernández-García, C., Dollar, F., Mancuso, C., Pérez-Hernández, J.A., Chen, M.-C., Hankla, A., Gao, X., Shim, B., Gaeta, A.L., Tarazkar, M., Romanov, D.A., Levis, R.J., Gaffney, J.A., Foord, M., Libby, S.B., Jaron-Becker, A., Becker, A., Plaja, L., Murnane, M.M., Kapteyn, H.C., Popmintchev, T.: Ultraviolet surprise: Efficient soft x-ray high-harmonic generation in multiply ionized plasmas. Science **350**(6265), 1225–1231 (2015) https://doi.org/10.1126/science.aac9755 https://www.science.org/doi/pdf/10.1126/science.aac9755

[3] Popmintchev, T., Chen, M.-C., Popmintchev, D., Arpin, P., Brown, S., Ališauskas, S., Andriukaitis, G., Balčiunas, T., Mücke, O.D., Pugzlys, A., Baltuška, A., Shim, B., Schrauth, S.E., Gaeta, A., Hernández-García, C., Plaja, L., Becker, A., Jaron-Becker, A., Murnane, M.M., Kapteyn, H.C.: Bright coherent ultrahigh harmonics in the kev x-ray regime from mid-infrared femtosecond lasers. Science **336**(6086), 1287–1291 (2012) https://doi.org/10.1126/science.1218497 https://www.science.org/doi/pdf/10.1126/science.1218497

[4] Vanroose, W., Martín, F., Rescigno, T.N., McCurdy, C.W.: Complete photo-induced breakup of the h¡sub¿2¡/sub¿ molecule as a probe of molecular electron correlation. Science **310**(5755), 1787–1789 (2005) https://doi.org/10.1126/science.1120263 https://www.science.org/doi/pdf/10.1126/science.1120263

[5] Delone, N.B., Krainov, V.P.: Tunneling and barrier-suppression ionization of atoms and ions in a laser radiation field. Physics-Uspekhi **41**(5), 469 (1998) https://doi.org/10.1070/PU1998v041n05ABEH000393

[6] Krause, J.L., Schafer, K.J., Kulander, K.C.: Optical harmonic generation in atomic and molecular hydrogen. Chemical Physics Letters **178**(5), 573–578 (1991) https://doi.org/10.1016/0009-2614(91)87022-4

[7] Christov, I.P.: Effects of spatial nonlocality versus nonlocal causality for bound electrons in external fields. Entropy **24**(6) (2022) https://doi.org/10.3390/e24060840

[8] Christov, I.P.: Dynamic correlations with time-dependent quantum monte carlo. The Journal of Chemical Physics **128**(24), 244106 (2008) https://doi.org/10.1063/1.2943674 https://pubs.aip.org/aip/jcp/article-pdf/doi/10.1063/1.2943674/15413573/244106$_{1o}$*nline.pdf*

[9] Chacón, A., Ciappina, M.F., Lewenstein, M.: Double-electron recombination in high-order-harmonic generation driven by spatially inhomogeneous fields. Phys. Rev. A **94**, 043407 (2016) https://doi.org/10.1103/PhysRevA.94.043407

[10] Koval, P., Wilken, F., Bauer, D., Keitel, C.H.: Nonsequential double recombination in intense laser fields. Phys. Rev. Lett. **98**, 043904 (2007) https://doi.org/10.1103/PhysRevLett.98.043904

[11] Hansen, K.K., Madsen, L.B.: Same-period emission and recombination in nonsequential double-recombination high-order-harmonic generation. Phys. Rev. A **93**, 053427 (2016) https://doi.org/10.1103/PhysRevA.93.053427

[12] Ammosov, M.V., Delone, N.B., Krainov, V.P.: Tunnel ionization of complex atoms and of atomic ions in an alternating electromagnetic field. Soviet Journal of Experimental and Theoretical Physics **64**(6), 1191 (1986)

[13] Wagner, N.L., Gibson, E.A., Popmintchev, T., Christov, I.P., Murnane, M.M., Kapteyn, H.C.: Self-compression of ultrashort pulses through ionization-induced spatiotemporal reshaping. Phys. Rev. Lett. **93**, 173902 (2004) https://doi.org/10.1103/PhysRevLett.93.173902

[14] Gibson, E.A., Paul, A., Wagner, N., Tobey, R., Backus, S., Christov, I.P., Murnane, M.M., Kapteyn, H.C.: High-order harmonic generation up to 250 ev from highly ionized argon. Phys. Rev. Lett. **92**, 033001 (2004) https://doi.org/10.1103/PhysRevLett.92.033001

[15] Cash, W.: Echelle spectrographs at grazing incidence. Appl. Opt. **21**(4), 710–717 (1982) https://doi.org/10.1364/AO.21.000710

[16] Li, J., Li, K., Zhang, X., Popmintchev, D., Xu, H., Yutong, W., Li, R., Zhang, G., Tang, J., niu, j., Ma, Y., Meng, R., Ke, C., Qiu, J., Ma, Y., Popmintchev, T., Fan, Z.: Highly efficient and aberration-free off-plane grating spectrometer and monochromator for euv—soft x-ray applications. Light: Science Applications **13** (2024) https://doi.org/10.1038/s41377-023-01342-9

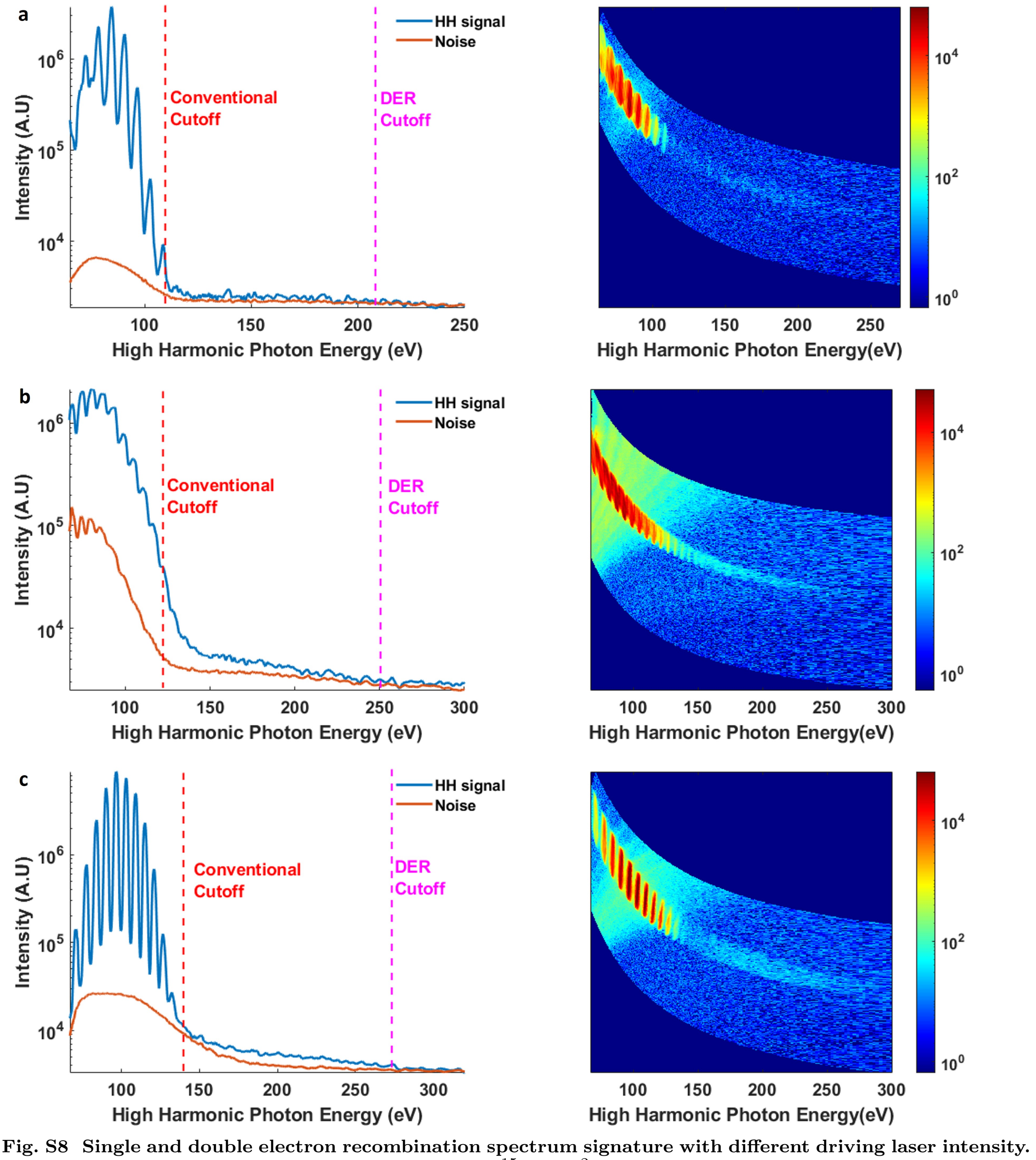

Fig. S8 Single and double electron recombination spectrum signature with different driving laser intensity. a) Driving laser intensity estimated to be around 1.7×10^{15} W/cm^2. The conventional cutoff is around 110 eV and the DER cutoff is around 210 eV. Which is the lowest intensity that we can start to observe the DER extended signal. b) Driving laser intensity estimated to be around 2.2×10^{15} W/cm^2. The conventional cutoff around 120 eV and DER cutoff around 250 eV. c) Driving laser intensity estimated to be around 2.6×10^{15} W/cm^2. The conventional cutoff is around 140 eV and the DER cutoff is around 280 eV.

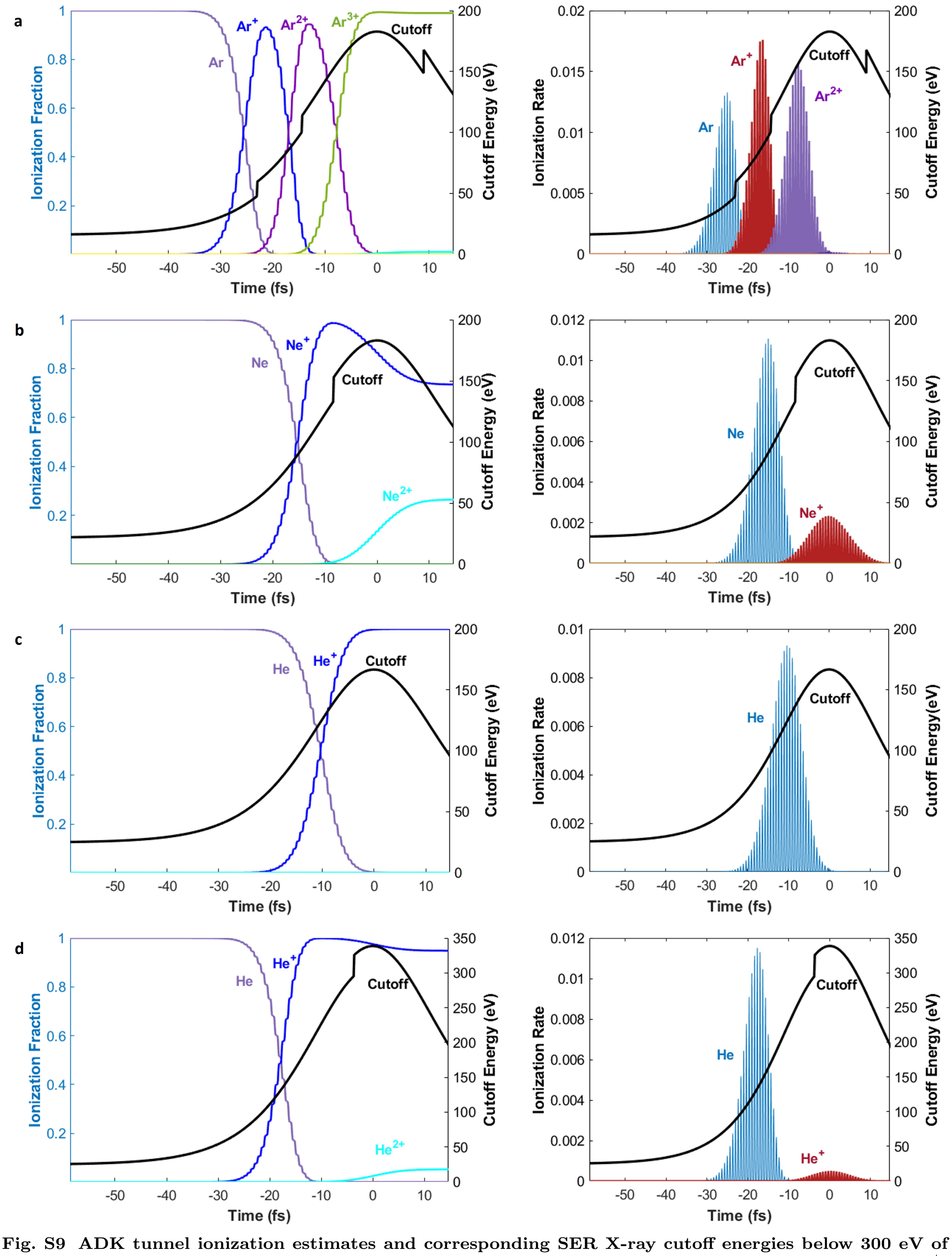

Fig. S9 ADK tunnel ionization estimates and corresponding SER X-ray cutoff energies below 300 eV of UV-driven high harmonic generation from atoms and ions. a-b) Tunnel ionization and SER cutoffs from Ar, Ar^{+}, Ar^{2+} and Ne, Ne^{+} by 400 nm 28 fs laser with intensity 3x10^{15} W/cm^2. c-d) Tunnel ionization from He and He^{+} by 400 nm 28 fs laser with intensity 3x10^{15} W/cm^2 and 6x10^{15} W/cm^2.

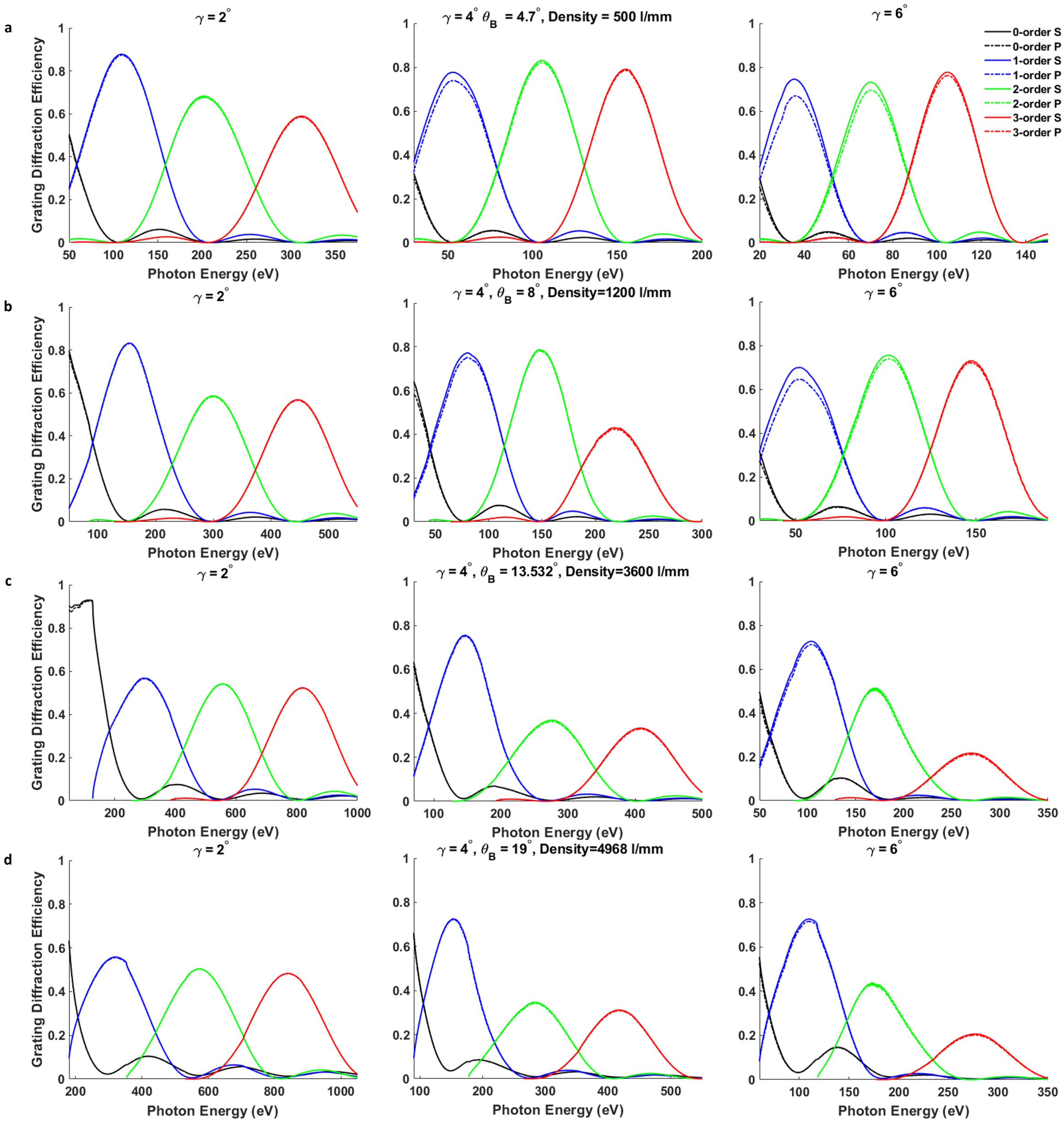

Fig. S10 Highly efficient conical-diffraction geometries for EUV and X-ray HHG detection with gratings of different groove and grazing incidence angles. Top to bottom: for increasing groove density and photon energy. **a)** 500 lines/mm with 4.7 degrees blazed angle. **b)** 1200 lines/mm with 8 degrees blazed angle. **c)** 3600 lines/mm with 13.5 degrees blazed angle. **d)** 4968 lines/mm with 19 degrees blazed angle. Left to right: for increasing incidence angles of 2°,4 °, and 6°.
