# Supplementary material for: Correlated electrons extend X-ray high-harmonic generation beyond the single-electron limit
Source: Nat Photonics. 2026 Aug 7;20(9):1092–8. doi: 10.1038/s41566-026-01976-2 (PMC13533847; doi:10.1038/s41566-026-01976-2)
Supplement: Supplementary file 2 [file 41566_2026_1976_MOESM2_ESM.pdf]

nature portfolio

Peer Review File

Correlated electrons extend X-ray high-harmonic generation beyond the single-electron limit

Corresponding Author: Dr Siyang Wang

This file contains all reviewer reports in order by version, followed by all author rebuttals in order by version.

Version 0:

Reviewer comments:

Reviewer #1

(Remarks to the Author)
The manuscript presents the observation of secondary plateau in high harmonic generation from a simplest strongly correlated electron system of Helium atom. The secondary plateau is not observed from Ar under similar conditions. Therefore based on the theoretical analysis the second plateau is attributed to the role of second electron recombination. The strong electron-electron correlations are expected in He but not in Ar. This is an important observation in terms of advancing the understanding of microscopic dynamics leading to generation of high harmonics. The manuscript is well-written and I support the publication if authors can address the following points in the revised manuscript.

1. What are the factors leading to exceptionally low conversion efficiency on the secondary plateau? It does not seem like just the quantum diffusion is able to explain this given the use of relatively short wavelength drive.

2. Is here a comparison experiment for the 800 nm drive. It is not clear why 400 nm is important if no crucial.

3. If figure 5 it might worth presenting data with 1d plot such that how the signal fall off as a function of ellipticity becomes more clear. From the model, is harmonic order dependence expected?

4. Observation of secondary plateau has been routine in solid-state materials as well as in liquids. It might be worth giving some context using these references: Nature volume 534, pages 520–523 (2016);https://doi.org/10.1038/s41467-017-00989-4; https://www.semanticscholar.org/reader/3c578d29673607a8305ab689c09516478e2cd14b;

Reviewer #2

(Remarks to the Author)
This is a very interesting paper opening new paths in High Harmonics Generation, AS the authirs argue "High-harmonic generation(HHG) underpins attosecond science. For over three decades, HHG has been framed within the confines of a single-active-electron model featuring a well-defined cutoff photon energy. Here, we demonstrate for the first time that correlated electrons can propel high-harmonic emission beyond this single-active-electron limit, dramatically extending the generated photon energies.
In this work, we observe a secondary plateau that pushes the cutoff beyond 100 eV into the water window. This phenomenon arises from the double-electron recombination among correlated electron pairs, resulting in a new cutoff scaling of up to 5.5 times the ponderomotive energy—significantly deviating from the conventional scaling of 3.2. These findings not only reshape our fundamental understanding of the HHG process but also reveal opportunities to expand the frontier of highharmonic sources. Furthermore, they position HHG as a potent photonic probe capable of unraveling attosecond correlations within quantum systems. Ultimately, our findings unlock new pathways in advanced ultrafast spectroscopy, innovative attosecond source development, and exploration into the realm of correlated quantum materials." I recommend acceptance.

Maybe in the outlook they could say that presented results and classical three step model theory call for quantum strong field approximation description and cite a review Symphony on Strong Field Approximation, Rep. Prog. Phys. 82, 116001 (2019), https://doi.org/10.1088/1361-6633/ab2bb1, (2019), arXiv:1812.11447.

Reviewer #3

(Remarks to the Author)
The authors report on the observation of a previously unknown plateau in the HHG spectrum of He with a cut-off energy of almost 2 times the one known from conventional HHG. This finding is attributed to the mutual action of two sequentially ionized electrons with their individually acquired energy
adding up upon recombination. The statement is tested against alternative explanations based on two major arguments:
1) The observed position of the extended DER cut-off is compatible with the result of a classical electron trajectory analysis including its dependence on the driving laser intensity (Fig.3). I consider this the most convincing one, although the underlying model disregards the true quantum nature of the process.
2) A variation of ellipticity of the driving laser field is stated to act more seriously on the 2nd plateau compared to the 1st one. This statement is less convincing as both plateaus in Fig. 5 vanish completely at decent ellipticity values and wether one or the other decreases more rapidly is hardly discernible in the log plot. Here, a more quantitative comparison should be presented. Finally, without comparison with a quantum mechanical simulation of the double-wavepacket evolution in the ionic continuum, this dependence remains a vague argument.
Still, the clarity of the observed feature warrants publication and the current lack of an advanced theoretical foundation will certainly trigger activity in the corresponding theory community. Correctly, the authors conclude, that '...the extended X-ray emission remains inherently weak due to low densities of correlated electron pairs...'. So the true value of this work is not to present a game changer for efficient water window harmonics (which the reader might expect from the abstract...), but rather 'a novel platform for probing femtosecond-to-attosecond electron-electron dynamics' This potential indeed justifies publication in Nature Photonics.

However, the authors may want to take a few further remarks into account:

A) It is stated that '...our results show clear evidence of high-harmonic emission from ionized argon species...the cutoff extension manifests as a shift of the main plateau to higher photon energies...'. Couldn't this shift be alternatively explained by operating the HHG process in the overdriven regime?

B) Page 6 reads ' In Fig. 5, asymmetries are observed between the signals driven by lasers with opposite spin polarizations'. What does 'spin polarizations' refer to? Photon spin? This would require circular polarization, but the asymmetry is observed for the two orthogonal linear polarization states...

C)In the supplementary material the captions for figs. S5 - S7 are mixed up.

Version 1:

Reviewer comments:

Reviewer #1

(Remarks to the Author)
Revised manuscript and the letter addresses my concerns adequately. My remaining suggestion is that if they could make a stronger connection between the atomic and solid state HHG physics in terms of the origin of the secondary plateau. This can help increase the impact of the paper to a larger set of audience.

The following are references related to the secondary plateau in solid-state HHG.
“Solid-state harmonics beyond the atomic limit.” Nature 534, 520–523 (2016), “High-harmonic generation in amorphous solids.” Nature Communications 8, 724 (2017), “Real-time observation of interfering crystal electrons in high-harmonic generation.” Nature 523, 572–575 (2015), “High-harmonic generation from Bloch electrons in solids.” Phys. Rev. A 91, 043839 (2015).
, “Enhancement of the second plateau in solid high-order harmonic spectra by the two-color fields.” Opt. Express 25, 18603 (2017).
DOI: https://doi.org/10.1103/PhysRevLett.129.167402 "Proposal for High-Energy Cutoff Extension of Optical Harmonics of Solid Materials Using the Example of a One-Dimensional ZnO Crystal", https://doi.org/10.1038/s41467-020-16480-6Attosecond science based on high harmonic generation from gases and solids

Reviewer #2

(Remarks to the Author)
The authors responded to all of the criticisms adequately. I suggest acceptance in Nature Photonics.

Reviewer #3

(Remarks to the Author)
The authors have adequately responded to my comments and have revised the manuscript accordingly.

I confirm my recommendation to publish in Nature Photonics.

Open Access This Peer Review File is licensed under a Creative Commons Attribution 4.0 International License, which permits use, sharing, adaptation, distribution and reproduction in any medium or format, as long as you give appropriate credit to the original author(s) and the source, provide a link to the Creative Commons license, and indicate if changes were made.
In cases where reviewers are anonymous, credit should be given to 'Anonymous Referee' and the source.
The images or other third party material in this Peer Review File are included in the article's Creative Commons license, unless indicated otherwise in a credit line to the material. If material is not included in the article's Creative Commons license and your intended use is not permitted by statutory regulation or exceeds the permitted use, you will need to obtain permission directly from the copyright holder.
To view a copy of this license, visit https://creativecommons.org/licenses/by/4.0/

Reviewer 1

The manuscript is well-written and I support the publication if authors can address the following points in the revised manuscript.

#Response#: We thank the referee for supporting the publication of our manuscript pending the revision of the listed points.

1. What are the factors leading to exceptionally low conversion efficiency on the secondary plateau? It does not seem like just the quantum diffusion is able to explain this given the use of relatively short wavelength drive.

#Response#: We thank the referee for this insightful question. Indeed, the driving wavelength is short, which leads to low quantum diffusion in the single-electron picture, thereby contributing to a high efficiency of harmonic generation within the single-electron-recombination cutoff. However, the presence of the secondary

plateau is attributed to an electron correlation mechanism. Our semi-classical trajectory analyses indicate that while one of the electrons follows a short trajectory, the other follows a longer trajectory. Additionally, it is important to note that the correlated electrons must satisfy a specific phase relationship to recombine with the parent ion simultaneously. Therefore, we believe the key factors influencing this phenomenon are as follows:

1. The low probability of forming correlated electron pairs with the constraint on the phase relationship between the two electrons to recombine simultaneously.

2. The quantum diffusion of the two-electron system is inherently more complex than that of a single-electron wavepacket scenario. For instance, in 3D, the diffusion of the electron wavepacket is increased by Coulomb repulsion in the transverse direction. In addition, higher-order rescattering of the wavepackets may lead to refocusing while returning 1 or 2 times to the core before recombination. If DER is understood as the reverse process of double ionization, then analogous to the lower probability of simultaneous double ionization, the probability of double recombination is also lower.

Future theoretical 3D simulations of the electron wavepackets versus 1D will help to reveal the dominant physics and quantify the efficiency of the secondary plateau.

3. The temporal phase-matching window is also critical, as the DER process requires a longer window due to the higher-order rescattering processes involved.

We have revised the main text in the 'Double Recombination and Electron Correlation Dynamics' section to emphasize these points more clearly. We thank the referee for bringing this to our attention.

2. Is here a comparison experiment for the 800 nm drive. It is not clear why 400 nm is important if no crucial.

#Response#: We have conducted experiments using an 800 nm driving wavelength in various similar configurations. Our investigations include both gas cell and fiber-based systems, as well as different spectrometer configurations, including the high-efficiency conical diffraction utilized in this study. In past work, we have also examined both over-driving regimes and optimal phase-matching conditions. Notably, an extended signal in the form of a secondary plateau with a step-wise sharp drop of efficiency has never been observed for high harmonic sources driven by 800 nm or longer mid-infrared wavelengths.

We believe that observing the secondary plateau with a short-wavelength UV driving laser is crucial for the detection and discovery of this phenomenon because:

Ionization levels and full phase matching: First, for high-harmonics driven by infrared or mid-infrared light, phase matching is more sensitive to laser-induced plasma density, with critical ionization for phase matching being below 5% for noble gases. In such scenarios, most of the experimentally observable signal comes from single-electron recombination, since macroscopic phase mismatch significantly decreases the signal from ions. Furthermore, quantum diffusion further decreases the signal level substantially under poor phase-matching conditions. The DER process requires a much larger ionization level to

The optimal temporal phase-matching window for an 800 nm high-harmonic source is usually below two driving laser cycles, which is insufficient to encompass the double-electron recombination process within the phase-matching window. This limitation prevents the formation and buildup of a possible secondary plateau. Effective phase matching using UV drivers can expand the temporal window for upconversion to more than 10 half laser cycles, resulting in a narrower linewidth of the harmonics.

Specifically, in the present work, a measured linewidth of sub-1 eV at FWHM within the SER cutoff up

to 130 eV, corresponding to a train of harmonic bursts in the time domain with an envelope of more than 1.82 fs at FWHM (assuming Gaussian profiles in both the time and frequency domains), or equivalently, an efficient phase-matching window of more than 3 half laser cycles. This facilitates the observation of high-order rescattering physics from a large ensemble of atoms. We included theoretical plots in the Supplemental Information illustrating the increase of the effective phase matching window over few laser cycles for UV-driven compared to IR-driven harmonics. Our estimates indicate the trend that for 400 nm UV drivers the effective phase matching window opens to 5 and 8 half laser cycles for He and Ar, while this window is limited to 2 and 3 half laser cycles for 800 nm driver.

At this stage of the experiments, we detect a weak DER plateau that appears as a quasi-continuum, indicating that the emission of these photons may originate from a near-single laser cycle. In fact, a narrow temporal window of DER emission can be expected because of the prerequisite of higher order returns spanning a few half laser cycles. Further experimental improvements and theoretical studies are needed to confirm this.

We have included figures for phase-matching in the supplementary materials(Fig.S3). They show the trend of broadening the effective phase-matching window with a shorter-wavelength driving laser(400 nm vs 800 nm). Even though this is not a precise phase-matching simulation, as the linear and nonlinear indices of refraction are not well known, the trend is significant and crucial for understanding the DER process.

We have revised the main text in the 'Double Recombination and Electron Correlation Dynamics' section to emphasize these points more clearly. We thank the referee for bringing this to our attention.

3. If figure 5 it might worth presenting data with 1d plot such that how the signal fall off as a function of ellipticity becomes more clear. From the model, is harmonic order dependence expected?

#Response#: We agree with the referee that a 1D plot of the yield as a function of driver ellipticity provides a clear picture of the dependence.

We have included a figure in the Supplementary Materials(Fig.S6) presenting the harmonics on a linear scale within both the conventional and secondary plateaus. Our measurements show that the harmonics in the two plateaus respond notably differently, with the secondary plateau being more sensitive to variations in ellipticity. From our experimental data, the harmonics within each plateau exhibit similar behavior. Based on these observations, we expect the underlying physics to occur within a few half-cycles, over which the electric field strength does not vary significantly.

The ellipticity dependence on harmonic order has been demonstrated at low harmonic orders within the conventional single-electron recombination plateau, where harmonics are generated over a broad temporal window[2]. In the DER process, however, the short time frame limits the contributing trajectories, and a specific phase relationship must be satisfied. In our measurements, no harmonic-order dependence is observed. While theory predicts such a dependence, it may be too subtle to resolve experimentally.

Future 3D quantum mechanical TDSE simulations of the wavepacket evolution in the continuum will certainly be needed to determine whether a harmonic-order dependence can be observed experimentally.

4. Observation of secondary plateau has been routine in solid-state materials as well as in liquids. It might be worth giving some context using these references: Nature volume 534, pages 520–523 (2016);https://doi.org/10.1038/s41467-017-00989-4; https://www.semanticscholar.org/

reader/3c578d29673607a8305ab689c09516478e2cd14b;

#Response#: Indeed, higher-density media such as liquids and solids routinely exhibit multiple plateaus. We have initiated theoretical and experimental studies of solids with the aim of identifying similar physical mechanisms and a unique spectral signature in ensembles with a large number of electrons and electron pairs, starting from gases and moving towards molecules and nanomaterials. We thank the referee for the suggestions; we have included the recommended literature and added a brief discussion in the 'Conclusions and Outlook' section.

Reviewer 2

1. This is a very interesting paper opening new paths in High Harmonics Generation, AS the authors argue.

#Response#: We would like to thank the reviewer for the beautiful comment and for recommending the manuscript for publication

.

2. Maybe in the outlook they could say that presented results and classical three step model theory call for quantum strong field approximation description and cite a review Symphony on Strong Field Approximation, Rep. Prog. Phys. 82, 116001 (2019), https://doi.org/10.1088/1361-6633/ab2bb1, (2019), arXiv:1812.11447.

#Response#: Thank you for the valuable suggestion! We agree that it is relevant to cite the rich review revealing valuable aspects of the physics of the process using SFA in the 'Conclusions and Outlook' section.

Reviewer 3

1. The observed position of the extended DER cut-off is compatible with the result of a classical electron trajectory analysis, including its dependence on the driving laser intensity (Fig.3). I consider this the most convincing one, although the underlying model disregards the true quantum nature of the process.

#Response#: This purely classical aspect of the calculation — involving single or two to three rescatterings of the electron before recombination — is identical to the second step of the semi-classical three-step model. The quantum nature of the process comes primarily into play through the correlations established during the ionization and recombination processes, where two electrons return simultaneously, emitting a single photon.

2. A variation of ellipticity of the driving laser field is stated to act more seriously on the 2nd plateau compared to the 1st one. This statement is less convincing as both plateaus in Fig. 5 vanish completely at decent ellipticity values and wether one or the other decreases more

rapidly is hardly discernible in the log plot. Here, a more quantitative comparison should be presented. Finally, without comparison with a quantum mechanical simulation of the double-wavepacket evolution in the ionic continuum, this dependence remains a vague argument.

#Response#: Both plateaus disappear at slightly different ellipticity values in our further data analysis. We strongly agree with the referee that representation on a linear scale will be more informative. We have included an additional figure in the supplementary materials comparing the intensity of selected harmonics in each plateau region as a function of laser ellipticity. Notably, the DER harmonics are more sensitive to the ellipticity of the driving laser compared to those of the SER harmonics.

We fully agree with the comment that the argument should be verified with a TDSE simulation of the two-electron wavepacket in the continuum. Developing a 3D rescattering model that accounts more realistically for quantum diffusion compared to a 1D model is essential. We thank the referee for this suggestion and have revised the text accordingly.

The disappearance of the signal at a relatively large ellipticity of the driving laser suggests that the process is a coherent rescattering process, as in conventional high-harmonic generation, rather than incoherent plasma radiation.

We have revised the main text in the 'Ellipticity Dependence of SER and DER High Harmonic Plateaus' section to emphasize these points more clearly. We thank the referee for bringing this to our attention.

Still, the clarity of the observed feature warrants publication and the current lack of an advanced theoretical foundation will certainly trigger activity in the corresponding theory community. Correctly, the authors conclude, that '...the extended X-ray emission remains inherently weak due to low densities of correlated electron pairs...'. So the true value of this work is not to present a game changer for efficient water window harmonics (which the reader might expect from the abstract...), but rather 'a novel platform for probing femtosecond-to-attosecond electron-electron dynamics' This potential indeed justifies publication in Nature Photonics.

#Response#: We greatly value the comment of the referee that the true value of the work is being potentially 'a novel platform for probing femtosecond-to-attosecond electron-electron correlation dynamics'. We appreciate the shared opinion that the results deserve publication in Nature Photonics. As the referee points out, the bright harmonics within the conventional plateau can be used as an ultra-bright coherent light source, while the harmonics in the secondary plateau could potentially serve as a photonic probe of strong electron correlations, at least qualitatively and possibly quantitatively.

Remark A. It is stated that '...our results show clear evidence of high-harmonic emission from ionized argon species...the cutoff extension manifests as a shift of the main plateau to higher photon energies...'. Couldn't this shift be alternatively explained by operating the HHG process in the overdriven regime?

#Response#: Yes, the shift of the conventional plateau of Ar to higher energy in the argon case can be well explained by the cutoff equation of the three-step model. As the driving laser intensity increases, the ponderomotive energy term increases. And at the same time, harmonics from different ions start to show up with higher ionization energy. For argon gas, we saw the first harmonics from argon atom, then harmonics

from Ar^+ ions, then harmonics generated from Ar^{2+} ions. Thus, both terms of the cutoff equation increase and conventional cutoff shifts to higher energy. However, there is no weaker extended secondary plateau observed with argon. Macroscopically, ideal phase matching is not possible at such high plasma levels for IR driving lasers; however, effective phase matching using UV lasers (where the higher indices of refraction of atoms and ions mitigate the plasma dispersion, as well as the single-atom yield increases) still leads to high signal levels. Notably, in this overdriven regime, the emission from atoms and ions is not substantially different. Earlier work reported high-harmonic spectra extending the conventional cutoff up to 500 eV in over-driven regimes from multiply-charged ions[3, 1, 4], yet without any secondary plateau characterized by a stepwise drop in yield of several orders of magnitude.

Remark B. Page 6 reads ' In Fig. 5, asymmetries are observed between the signals driven by lasers with opposite spin polarizations'. What does 'spin polarizations' refer to? Photon spin? This would require circular polarization, but the asymmetry is observed for the two orthogonal linear polarization states...

#Response#: We would like to clarify that the correct term here is driving laser polarization. 'Spin polarization' is an incorrect term in this context. We mention in the text that an asymmetry is observed between the signals driven by lasers with orthogonal linear polarization(s- and p-). To elaborate, such an asymmetry is anticipated as there is a Brewster window at the entrance of our vacuum beamline that optimizes the transmission of p-polarized light. Downstream, both the toroidal mirror and the conical diffraction grating respond slightly differently to s- and p-polarized light, which further amplifies the asymmetry.

We thank the referee for this remark. We have clarified the caption based on the suggestion in the main text and in the supplementary materials. We appreciate your time for careful reading!

Remark C. In the supplementary material, the captions for figs. S5 - S7 are mixed up.

#Response#: We apologize for the mistake in editing. We fixed the mixed-up captions. Again, we do appreciate your careful reading!

References

[1] P. Arpin et al. "Enhanced High Harmonic Generation from Multiply Ionized Argon above 500 eV through Laser Pulse Self-Compression". In: *Phys. Rev. Lett.* 103 (14 Sept. 2009), p. 143901. doi: 10.1103/PhysRevLett.103.143901. url: https://link.aps.org/doi/10.1103/PhysRevLett.103.143901.

[2] K. S. Budil et al. "Influence of ellipticity on harmonic generation". In: *Phys. Rev. A* 48 (5 Nov. 1993), R3437–R3440. doi: 10.1103/PhysRevA.48.R3437. url: https://link.aps.org/doi/10.1103/PhysRevA.48.R3437.

[3] Emily A. Gibson et al. "High-Order Harmonic Generation up to 250 eV from Highly Ionized Argon". In: *Phys. Rev. Lett.* 92 (3 Jan. 2004), p. 033001. doi: 10.1103/PhysRevLett.92.033001. url: https://link.aps.org/doi/10.1103/PhysRevLett.92.033001.

[4] Dimitar Popmintchev et al. "Ultraviolet surprise: Efficient soft X-ray high-harmonic generation in multiply ionized plasmas". In: *Science* 350 (Dec. 2015), p. 1225. doi: 10.1126/science.aac9755.

Authors Response to Reviews

June 20, 2026

Dear Editors and Reviewers of Nature Photonics NPHOT-2025-08-0186,

We would like to express our sincere appreciation for the time and consideration you devoted to our manuscript. We are encouraged by the positive indications from the review process and are grateful for the constructive feedback that helped strengthen the work.

Thank you for the opportunity to contribute to Nature Photonics and for facilitating a rigorous and thoughtful review process.

Sincerely,

Siyang Wang
On Behalf of All Authors

Reviewer 1

Revised manuscript and the letter addresses my concerns adequately. My remaining suggestion is that if they could make a stronger connection between the atomic and solid state HHG physics in terms of the origin of the secondary plateau. This can help increase the impact of the paper to a larger set of audience.

#Response#: We thank the reviewer for this insightful suggestion. We agree that explicitly connecting atomic and solid-state HHG physics regarding the origin of the secondary plateau significantly broadens the appeal and impact of our work.To address this, we have revised the relevant paragraph in the "Conclusion and Outlook" section that directly discusses this bridge.

The following are references related to the secondary plateau in solid-state HHG. "Solid-state harmonics beyond the atomic limit." Nature 534, 520–523 (2016), "High-harmonic generation in amorphous solids." Nature Communications 8, 724 (2017), "Real-time observation of interfering crystal electrons in high-harmonic generation." Nature 523, 572–575 (2015), "High-harmonic generation from Bloch electrons in solids." Phys. Rev. A 91, 043839 (2015). , "Enhancement of the second plateau in solid high-order harmonic spectra by the two-color

fields." Opt. Express 25, 18603 (2017). DOI: https://doi.org/10.1103/PhysRevLett.129.167402 "Proposal for High-Energy Cutoff Extension of Optical Harmonics of Solid Materials Using the Example of a One-Dimensional ZnO Crystal", https://doi.org/10.1038/s41467-020-16480-6Attosecond science based on high harmonic generation from gases and solids.

#Response#: We sincerely thank the reviewer for recommending these excellent references. We entirely agree that these papers provide vital context and beautifully bridge the gap between atomic and solid-state HHG physics regarding the secondary plateau. Consequently, we have incorporated all seven suggested references into our revised discussion within the "Conclusion and Outlook" section (now cited as Refs. [46-51] and [53].).

Reviewer 2

The authors responded to all of the criticisms adequately. I suggest acceptance in Nature Photonics.

#Response#: We sincerely thank the reviewer for their time, constructive feedback throughout the peer-review process, and their recommendation for acceptance in Nature Photonics. We are very pleased that our revisions have adequately addressed all previous concerns.

.

Reviewer 3

The authors have adequately responded to my comments and have revised the manuscript accordingly. I confirm my recommendation to publish in Nature Photonics.

#Response#: We are very grateful to the reviewer for their careful evaluation of our manuscript throughout the review process and for confirming their recommendation for publication in Nature Photonics. We appreciate their valuable feedback, which helped improve the quality of our work.
